# Breast cancer stromal clotting activation (Tissue Factor and thrombin): A pre‐invasive phenomena that is prognostic in invasion

**DOI:** 10.1002/cam4.2748

**Published:** 2020-01-21

**Authors:** Hudhaifah Shaker, Nigel J. Bundred, Göran Landberg, Susan A. Pritchard, Harith Albadry, Sarah L. Nicholson, Lauren J. Harries, Jing Y. E. Heah, John Castle, Cliona C. Kirwan

**Affiliations:** ^1^ Faculty of Biology, Medicine and Health Division of Cancer Sciences School of Medical Sciences Manchester Cancer Research Centre University of Manchester Manchester UK; ^2^ Department of Pathology Institute for Biomedicine Sahlgrenska Cancer Center University of Gothenburg Gothenburg Sweden; ^3^ Department of Histopathology Manchester University NHS Foundation Trust Wythenshawe, Manchester UK; ^4^ Department of Histopathology Royal Liverpool and Broadgreen University Hospitals NHS Trust Liverpool UK; ^5^ Department of Histopathology East Lancashire Hospitals NHS Trust Blackburn UK; ^6^ The Nightingale Centre and Prevent Breast Cancer Research Centre Wythenshawe Hospital Manchester University NHS Foundation Trust Manchester UK

**Keywords:** breast cancer, coagulation, DCIS, fibroblast, PAR1, PAR2, thrombin, thrombosis, tissue factor

## Abstract

**Background:**

Tumor stroma, of which fibroblasts are the most abundant cell, resembles a non‐healing wound, where a procoagulant environment creates a permissive milieu for cancer growth. We aimed to determine if tumor expression of coagulation factors (procoagulant phenotype), and systemic hypercoagulability, occur at the preinvasive (ductal carcinoma in situ; DCIS) stage and correlate with breast cancer subtype, disease‐free survival (DFS), and overall survival (OS).

**Methods:**

In a prospective cohort of early breast cancer (DCIS, n = 76; invasive, n = 248) tumor, normal breast and plasma were examined. Fibroblast and epithelial expression of Tissue Factor (TF), thrombin, PAR1, PAR2, and plasma thrombin‐antithrombin (TAT) and D‐dimer were correlated with clinicopathological data, and 5‐year survival.

**Results:**

Fibroblast expression of TF, thrombin, and PAR1 was increased in DCIS and invasive cancer compared to normal breast fibroblasts (*P* ≤ .003, all). Fibroblast TF, thrombin, PAR1, and PAR2 was increased in cancers with high Ki67, high grade, ER‐ (vs ER+), and HER2+ (vs HER2‐) (all *P* < .05). On univariate analysis, fibroblast TF expression was inversely associated with DFS (*P* = .04) and OS (*P* = .02). D‐dimer was higher in node positive (507 (CI: 411‐625) ng/mL, n = 68) vs negative patients (428 (CI: 387‐472) ng/mL, n = 171, *P* = .004) and inversely associated with OS (*P* = .047). On multivariate analysis, plasma TAT was associated with reduced OS (HR 3.26, CI 1.16‐3.1, *P* = .02), with a high plasma TAT (≥3.2 ng/mL) associated with > 3‐fold mortality risk compared to low TAT.

**Conclusion:**

This demonstrates procoagulant phenotypic changes occur in fibroblasts at the preinvasive stage. Fibroblast procoagulant phenotype is associated with aggressive breast cancer subtypes and reduced survival. Coagulation may be a therapeutic target in breast cancer.

## Graphical abstract

In a large prospective cohort study (n = 324) of breast cancer patients, increased expression of markers of coagulation (Tissue Factor, thrombin and their respective receptors PAR2 and PAR1) are localized to fibroblasts, increased in aggressive breast cancer subtypes, and correlate with reduced overall survival. The phenotypic change in fibroblasts to a procoagulant state occurs at the preinvasive (DCIS) stage, implying an epithelial‐stromal communication across the basement membrane in DCIS creates a procoagulant stromal milieu and potentially an environment to facilitate invasion.

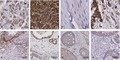

## 1 INTRODUCTION

There were over 2 million new breast cancer cases worldwide in 2018.^1^ It is the commonest cause of female cancer death, highlighting that new treatment strategies are required.

Tumor stroma is a potential target for novel treatments. Gene expression studies show that in transitioning from benign through pre‐invasion (DCIS) to invasive breast cancer, tumor stroma undergoes changes paralleling the evolution of gene expression in the malignant epithelium.^2^ Within invasive tumor stroma, cancer‐associated fibroblasts (CAFs) are key promoters of tumor development^3^ and growth.^4^ Stromal expression of CAF markers correlate with reduced survival^5^ with stromal gene expression predicting outcome in breast cancer.^6^ In vivo CAFs appear important in promoting transition from DCIS to invasion.^7^

Tumor stroma resembles a “wound that will not heal.”^8^ The primary phase of wound healing is leakage of extrinsic clotting pathway factors and blood components to instigate clot formation. This procoagulant environment creates a permissive milieu for extravasation, extracellular remodelling, cell differentiation and angiogenesis.

Cancer and coagulation have a symbiotic relationship. Cancer induces a hypercoagulable state,^9^ presenting clinically with an almost fivefold increased incidence of venous thromboembolism (VTE).^10^ Conversely the extrinsic clotting pathway promotes cancer progression. Tissue Factor (TF) is an integral membrane protein and the primary initiator of the extrinsic clotting pathway, converting prothrombin to thrombin, thus inducing hemostasis. TF promotes tumor growth, migration, and angiogenesis, independent of the coagulation cascade, via activation of protease‐activated receptor (PAR)2.^11^ Thrombin promotes tumor growth, angiogenesis, and metastasis through PAR1^12^, ^13^ and through fibrin generation, for example by shielding tumor cells from immune surveillance.^14^ We hypothesize that in tumor stroma a process similar to wound healing occurs, with TF and its downstream effector molecules promoting cancer progression.

In early breast cancer patients with DCIS or invasive disease, we aimed to investigate whether immunohistochemistry determined stromal fibroblast expression of procoagulants (TF, thrombin, PAR1, and PAR2) are related to breast cancer phenotype, clinicopathological predictors of outcome, systemic markers of hypercoagulability and survival.

## 2 MATERIALS AND METHODS

A prospective cohort (CHAMPion study: Cancer Induced Hypercoagulability As a Marker of Prognosis, UKCRN ID:8685) of treatment‐naïve early breast cancer patients (DCIS and invasive) undergoing surgical resection were recruited at Manchester University NHS Foundation Trust (MFT) between August 2010 and July 2014. Venous blood was collected prior to surgery. FFPE tumor and normal tissue distant from the tumor was sampled. CHAMPion was conducted in line with STROBE and REMARK statements (Appendix A).

The CHAMPion study was approved by Oldham Research Ethics Committee (Ref:09/H1011/47) and sponsored by MFT (R&D Ref:2010SG001).

### 2.1 Tissue analysis

Tissue microarrays (TMAs) were constructed using a tissue arrayer (MTA‐01, Beecher Inc, WI). Immunohistochemistry (IHC) was performed with Dako's Autostainer Plus (K8010, Dako) using commercial antibodies (methodology including antibody validation, Appendix B). Two 1 mm diameter tissue samples per patient were manually dual‐scored by independent scorers (SP, HA, SN, LH) blinded to clinical and pathological information.

**Figure 1:**
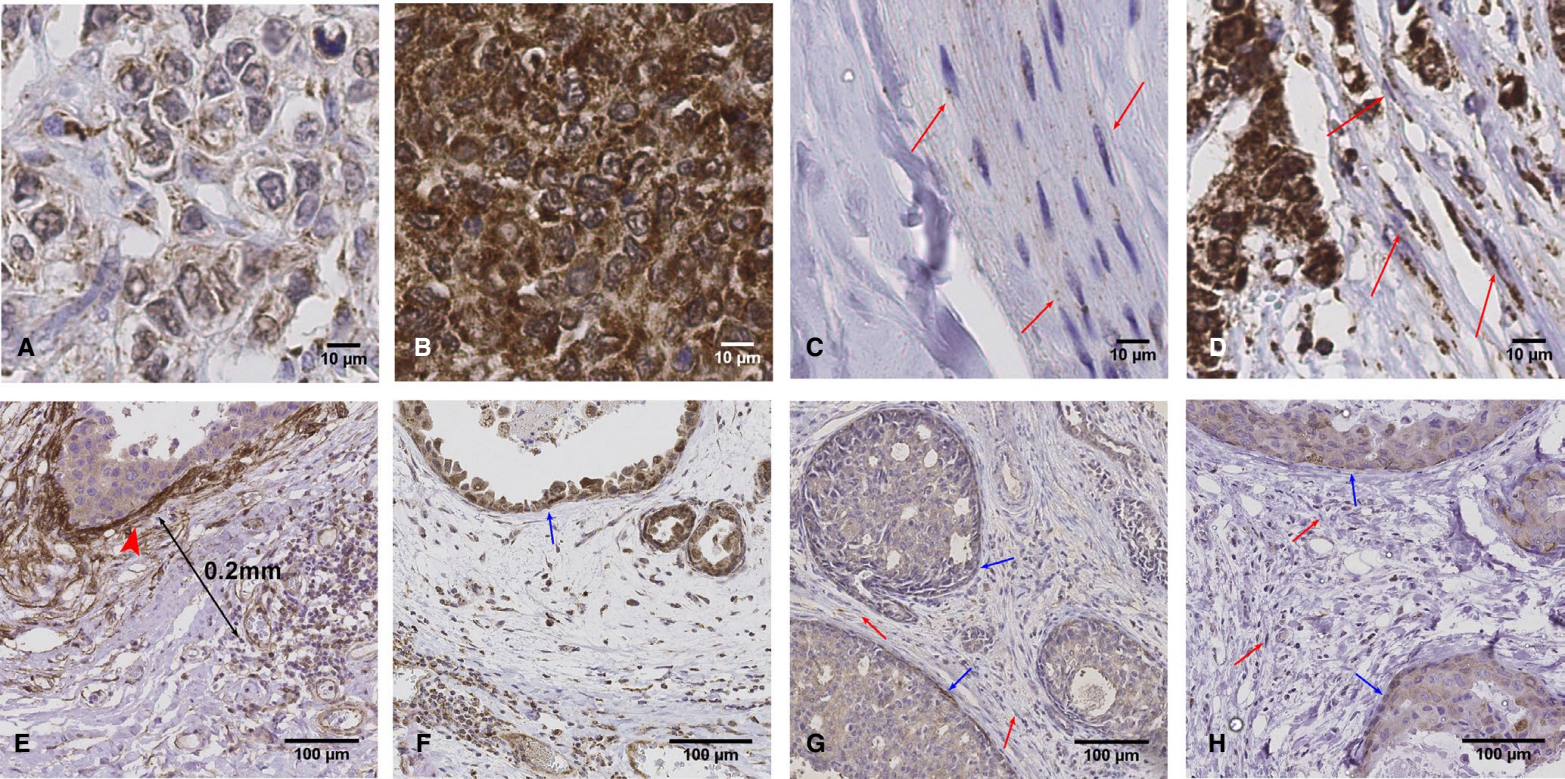
Tissue Microarray scoring of epithelial and stromal fibroblast extrinsic clotting pathway expression. Row 1: Thrombin epithelial staining: Images of invasive breast cancer tissue Immunohistochemistry (IHC)‐stained for thrombin with (A) low and (B) high epithelial staining. Row 1: Thrombin stromal staining and fibroblast scoring: Images of tissue IHC‐stained for thrombin with (C) <20% of stromal fibroblasts exhibiting staining and (D) 80% of stromal fibroblasts exhibiting staining. Example fibroblasts are pointed out with red arrows. Row 2: Tissue Factor (TF), thrombin, PAR1, and PAR2 ductal carcinoma in situ (DCIS) fibroblast scoring. DCIS tissue IHC stained for (E) Tissue Factor (F) thrombin (G) PAR1 (H) PAR2. In general, TF demonstrated strong myoepithelial staining (arrowhead, figure E). To avoid the potential risk of overscoring fibroblast TF staining, through including myoepithelial cells, TF fibroblast scoring was performed at a minimum of 0.2 mm away from the duct (Figure 1E). Intense myoepithelial staining was not a feature of thrombin, PAR1 or PAR2 IHC stains. (Blue arrows: myoepithelial cells, red arrows: fibroblasts)

Tissue Factor, PAR1, PAR2, and Thrombin showed predominantly cytoplasmic and membranous staining with no nuclear expression seen. This was the case for normal and cancer epithelium and fibroblasts. Epithelial expression was predominantly homogeneous within tumor samples (ie, most epithelial cells stained in a bimodal fashion, being either all negative or all positive on a particular whole slide or 1 mm section). The intensity of staining did vary between cases. Therefore, epithelial expression was scored on a scale of 0‐3 by intensity alone (and not percentage of cell) where 0 is negative, 1 is weakly positive (~100% of cells are weakly positive), 2 is moderately positive, and 3 is strongly positive. (Figure 1A,B; Figure C.1‐3).

Stromal TF, thrombin, PAR1, and PAR2 staining scoring was restricted to cells morphologically consistent with fibroblasts.^15^ There was variation in the percentage of fibroblasts that showed cytoplasmic or membranous expression within a whole slide or 1 mm section, but with little variation in intensity. Fibroblast expression was scored on the basis of the percentage of fibroblasts that showed any expression to the nearest 20% of all visible fibroblasts in a 1 mm section. This was done for two specimens per sample (normal/cancer 1 mm core sections) and a mean calculated for each. Data for each marker was expressed as a continuous variable and dichotomised into high and low based upon the median for that marker. The median percentage fibroblast expression was 80% for TF, 70% for Thrombin, 60% for PAR1, and 60% for PAR2 (Figure 1C,D).

In normal and DCIS tissue, thrombin, PAR1, and PAR2 fibroblast staining was diffusely spread throughout the stroma. TF stromal staining was particularly concentrated around ducts, reflecting previously reported myoepithelial cell TF expression.^16^ To ensure TF fibroblast scoring was independent of strong myoepithelial staining, in normal tissue and DCIS, only fibroblasts > 0.2 mm from ducts were scored (Figure 1E). All fibroblasts were included in thrombin, PAR1, and PAR2 scorings (Figure 1F‐H).

Tumor size, grade, estrogen receptor (ER) (invasive and DCIS) and lymph node, HER2, Ki67 (invasive) were determined as per UK National Health Service Breast Screening Programme Guidelines. ER, HER2, and Ki67 were dichotomized (ER‐positive: Quick Score ≥ 3; HER2‐positive: IHC score 3+ or amplified on in situ Hybridization; Ki67 high: >20%).

### 2.2 Statistical analysis

The study was powered to identify preoperative plasma D‐dimer as a biomarker of lymph node metastases. Of 250 invasive breast cancer patients, approximately 30% were expected to be node‐positive, giving 90% power to detect differences of 26% or more in the percentage with raised D‐dimer (>500 ng/mL) between node‐positive and node‐negative patients (60% vs 34%).

Means were expressed ± standard error (SE). Student's *t* test and analysis of variance (ANOVA) compared continuous variables. Fisher's Least Significant Differences test compared groups following ANOVA. Categorical and continuous variables were compared using Chi‐squared and Spearman's rank correlation coefficient (CC), respectively.

The tissue analyses were comparison of fibroblast TF/thrombin/PAR1/PAR2 expression in normal tissue compared to DCIS and to invasive breast cancer; and correlation of fibroblast TF/thrombin/PAR1/PAR2 expression with pathological predictors of outcome, clinical outcome at median 5 years and systemic hypercoagulability.

The association between survival (DFS or OS) and clinicopathological variables and procoagulant markers (tissue and plasma) was assessed using univariate Cox proportional hazards, with significant variables entered in to a multivariate model following backward stepwise selection (Appendix D).

Differences were considered significant if *P* ≤ .05. SPSS 20.0 for windows (IBM Corp. NY) was used.

## 3 RESULTS

Of the 762 patients screened, 324 (invasive breast cancer, n = 248: DCIS, n = 76) were included in the final analysis (Figure A.1). Clinicopathological details are provided in Table 1. Median follow‐up from diagnosis was 62 (range 8‐80) months. All‐cause mortality (invasive patients only) was 6% (n = 15) and 13 patients (5.2%) developed recurrent disease (local or distant).

**Table 1:** Clinicopathological details for DCIS and invasive cancer patients

	DCIS (n = 76) n (%)^a^	Invasive cancer (n = 248) n (%)^a^
Age(years)^b^	60 (32‐79)	59 (24‐84)
Presentation		
Symptomatic	7 (12)	73 (31)
Screening	49 (88)	166 (69)
Invasive cancer pathology^c^		
Invasive ductal		219 (90)
Invasive lobular		21 (9)
Other		3 (1)
Tumour size, mm		
Mean (range)	31.7 (1‐150)	16.4 (1‐70)
Grade^c^ (DCIS/ invasive)		
Low/1	1 (1)	63 (26)
Intermediate/2	18 (25)	104 (42)
High/3	54 (74)	78 (32)
ER status^c^		
Positive	50 (68)	202 (82)
Negative	23 (32)	44 (18)
PR status^c^		
Positive	41 (56)	170 (69)
Negative	32 (44)	76 (31)
Microinvasion^c^		
Yes	6 (9)	
No	65 (81)	
HER2 receptor status^c^		
Positive		27 (11)
Negative		219 (89)
Ki67 expression^c^		
<20%		130 (55)
>20%		105 (45)
Lymph node status^c^		
Positive		68 (28)
Negative		177 (72)
Disease recurrence		
Yes		13 (5.2)
No		233 (94.8)
Disease free survival (years)		
Mean (95% CI)		59.7 years (1.8‐76.8)
Died during follow up		
Yes		15 (6)
No		231 (94)
Overall survival (years)		
Mean (95% CI)		61.7 years (8.4‐80.2)

a Unless otherwise stated

b Median (range)

c Data unavailable on some patients;

### 3.1 Epithelial expression of thrombin is increased in invasive breast cancer

*Epithelial* expression of thrombin was higher in invasive cancer compared to DCIS (45% vs 22%, *P* = .01) and compared to normal breast tissue (45% vs 16%, *P* < .001). However, there was no difference in the *epithelial* expression of TF, PAR1, or PAR2 among normal breast tissue, DCIS, or invasive cancer (Table E.1).

### 3.2 A cancer‐like procoagulant fibroblast phenotype develops preinvasion

**Figure 2:**
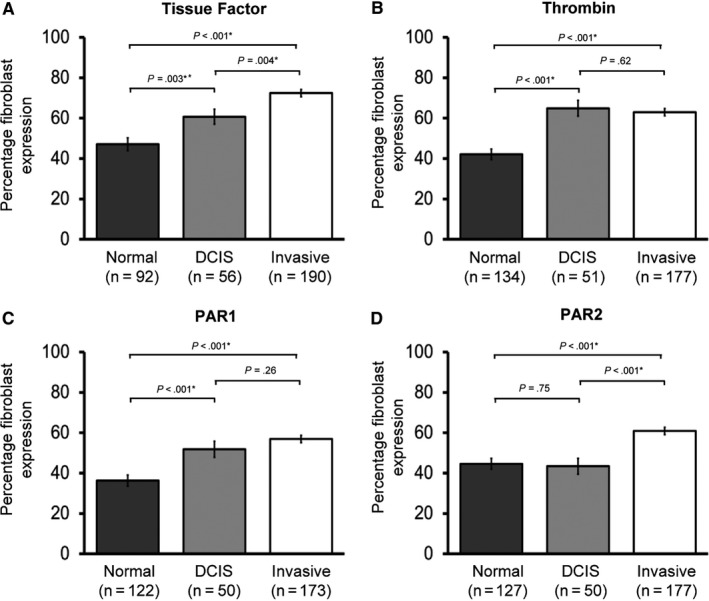
Stromal expression of the extrinsic clotting pathway is increased in ductal carcinoma in situ (DCIS) and invasive breast cancer. Fibroblast expression of (A) Tissue Factor, (B) thrombin, (C) PAR1, and (D) PAR2 in the CHAMPion cohort is shown. Data is presented as mean percentage fibroblasts with positive expression ± Standard error of the mean (SEM). Number of samples tested shown in brackets. Statistical differences between groups were tested using ANOVA and Fisher's Least Significant Differences test for post hoc analyses. Normal, normal breast tissue; DCIS, ductal carcinoma in situ tissue; Invasive, invasive breast cancer tissue

Fibroblast TF, thrombin, PAR1, and PAR2 were increased in invasive cancer compared to normal breast tissue (*P* < .001, all) (Figure 2, Table E.2). However, even in DCIS, fibroblast expression of TF, thrombin, and PAR1 was increased compared to normal breast fibroblasts (*P* ≤ .003). This implies procoagulant phenotypic evolution of fibroblasts is a preinvasive phenomenon, highlighting that a procoagulant stroma may have a functional role in promoting transition to invasion.

### 3.3 Fibroblast procoagulant expression is increased in aggressive breast cancer subtypes

**Figure 3:**
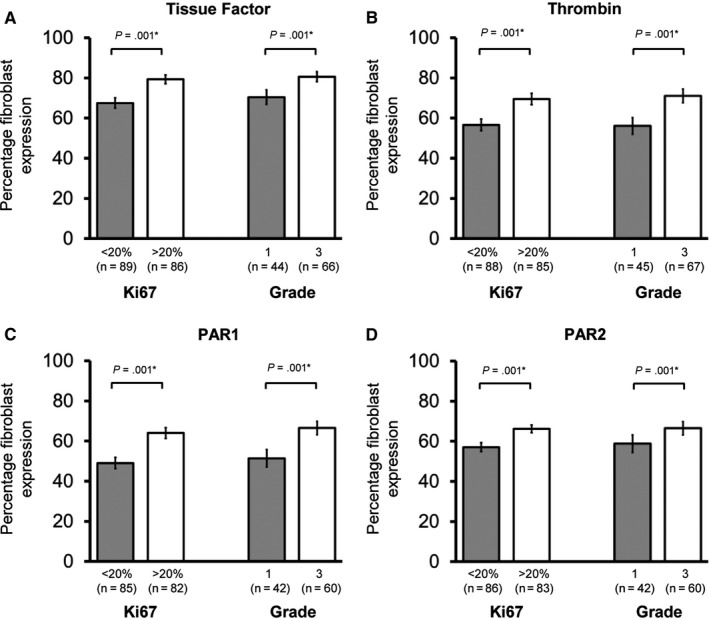
Fibroblast expression of the extrinsic clotting pathway is increased in high proliferation and high‐grade invasive breast cancers. Fibroblast expression of (A) Tissue Factor, (B) thrombin, (C) PAR1, and (D) PAR2 according to Ki67 expression and grade (1 vs 3) in invasive cancer patients is shown. Data are presented as mean percentage fibroblasts with positive expression ± Standard error of the mean (SEM). Number of samples tested shown in brackets. Statistical differences between groups were tested using Student's *t* test

**Figure 4:**
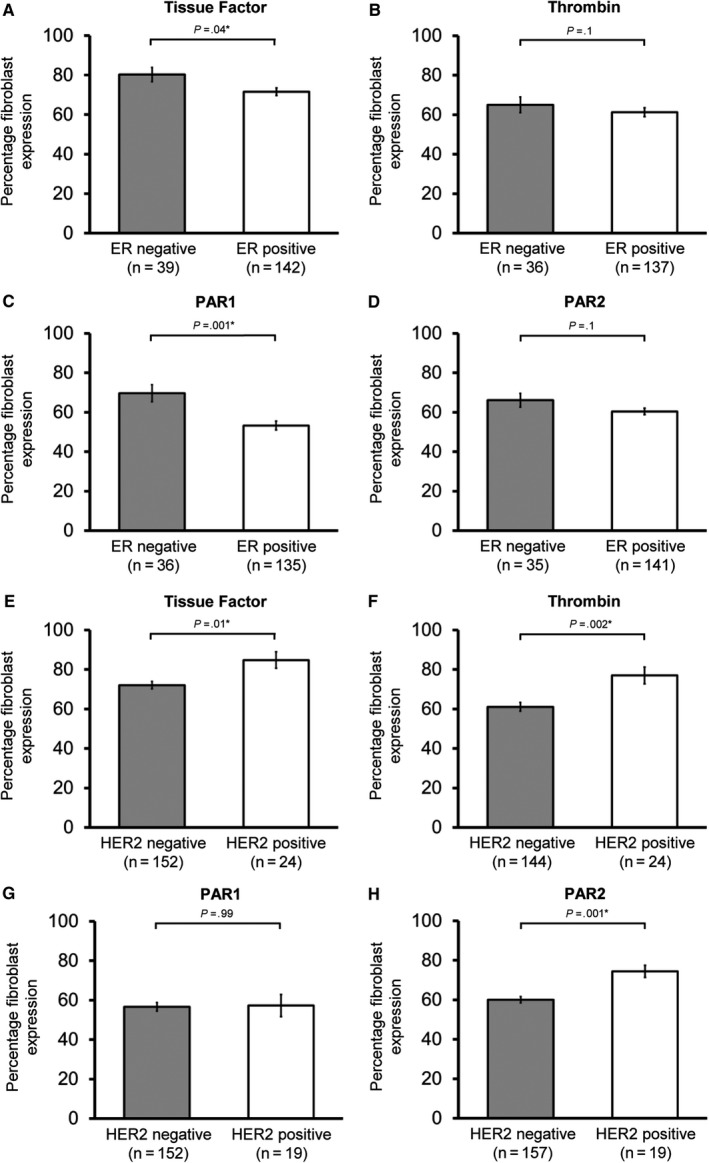
Fibroblast expression of the extrinsic clotting pathway is increased in estrogen receptor (ER)‐negative and HER2‐positive breast cancer. Fibroblast expression of (A) Tissue Factor, (B) thrombin, (C) PAR1, and (D) PAR2 in ER‐negative and ER‐positive cancers and (E) Tissue Factor, (F) thrombin, and (G) PAR2 in HER2‐negative and HER2‐positive cancers is shown. Data are presented as mean percentage fibroblasts with positive expression ± Standard error of the mean (SEM). Number of samples tested shown in brackets. Statistical differences between groups were tested using Student's *t* test. ER, oestrogen receptor. HER2, Human epidermal growth factor receptor 2

Fibroblast expression of TF, thrombin, PAR1, and PAR2 were increased in cancers with high Ki67 (all *P* ≤ .002, Figure 3) and higher grade disease (grade 1 vs 3, all *P* < .001, Figure 3). ER‐negative invasive breast cancer was associated with increased fibroblast expression of TF (*P* = .04), and PAR1 (*P* = .001), with possible trends in thrombin and PAR2 (both *P* = .1, Figure 4). HER2 positivity was associated with increased fibroblast expression of TF (*P* = .01), thrombin (*P* = .002), and PAR2 (*P* = .001, Figure 4). In relation to intrinsic subtypes based on immunohistochemical data, there was a trend toward greater fibroblast expression of extrinsic pathway markers in Luminal B (ER positive/ Her2 negative/ Ki67 high) vs Luminal A (ER positive/ Her2 negative/ Ki67 low) and in HER2‐postive and Basal (ER/PR/HER2 negative) versus Luminal cancers. However, lower number of HER2‐positive and Basal cancers compared to Luminal cancers limited interpretation of results (Appendix E).

There was no association between fibroblast procoagulant markers and tumor size. PAR1 fibroblast expression was increased in node positive cancers (66% vs 54%, *P* = .005), with PAR2 (66% vs 60%, *P* = .08) demonstrating a similar trend (Appendix E).

In invasive cancer, on univariate analysis, fibroblast TF expression was associated with reduced OS (*P* = .02) and DFS (*P* = .04).

On multivariate analysis, fibroblast TF expression demonstrated a possible association with reduced OS, with a 1% increase in fibroblast TF expression equating to a 3.8% increase HR for all‐cause mortality (HR 1.038, CI: 0.99‐1.08, *P* = .09, Fig. D.1). This equates to TF fibroblast expression of 60% intensity having twice the mortality risk compared to expression of 40% intensity.

### 3.4 Fibroblast procoagulant expression in DCIS

Thrombin expression was increased in ER‐negative (n = 17) compared to ER‐positive (n = 27) DCIS (71.8%, S.E 5.3% vs 57.0%, S.E 4.8; *P* = .05) demonstrating the association between procoagulant stroma with poorer prognosis disease at the preinvasive stage. There was no association between DCIS grade or size and stromal expression of TF, thrombin, PAR1, or PAR2 (Appendix E).

### 3.5 Plasma coagulation markers correlate with lymph node positivity

Pre‐operative TF, TAT, and D‐dimer all correlated (CC 0.11‐0.13, *P* ≤ .05). Plasma markers of coagulation did not differentiate between DCIS and invasive patients, or correlate with ER, HER2, Ki67, tumor size, or grade (Appendix F).

However, D‐dimer was higher in node positive invasive patients (node positive [n = 68]: geometric mean 507 (CI 411‐625) ng/mL; node negative [n = 171]: 428 (CI 387‐472) ng/mL, *P* = .004). Using the clinical definition of raised D‐dimer (>500 ng/mL), pre‐operatively 50% of node positive patients had raised D‐dimer, compared to 35% of node negative patients (*P* = .03). On multivariate analysis, D‐dimer > 559 ng/mL (determined by Receiver Operating Characteristic curve) predicted node positivity (OR 2.53, CI:1.33‐4.83, *P* = .005, Figure F.1).

**Figure 5:**
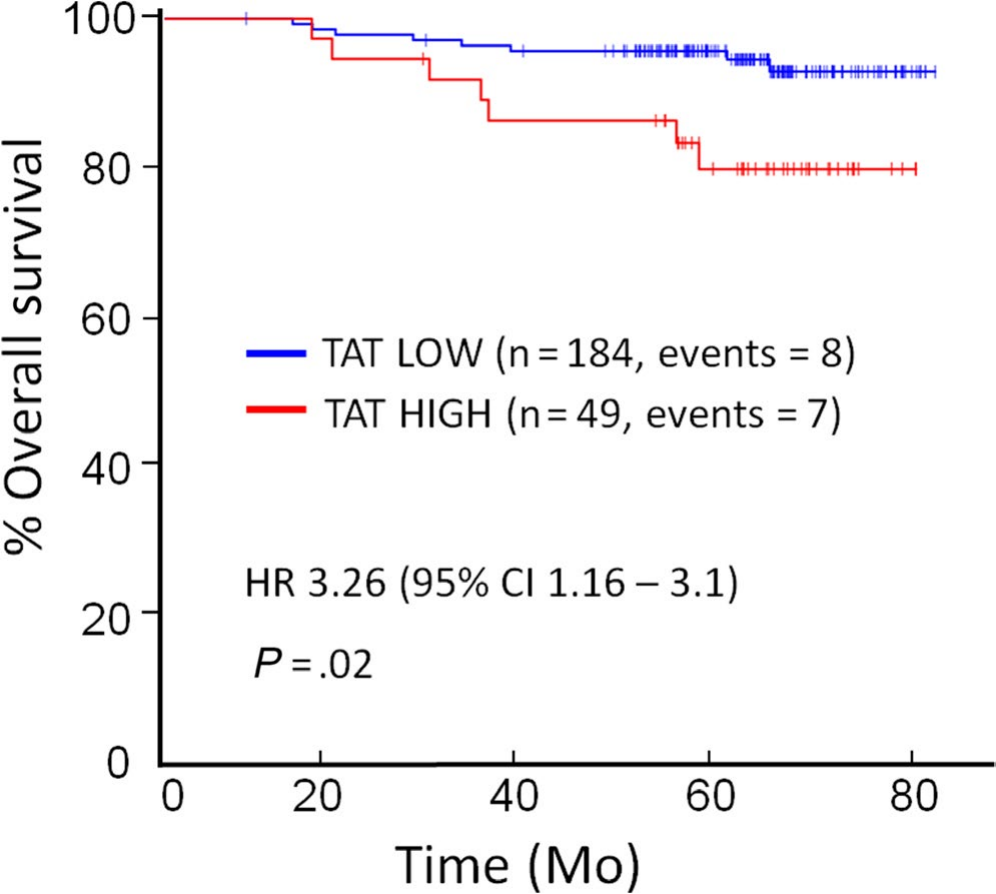
Kaplan‐Meier survival curves for overall survival according to plasma thrombin‐antithrombin (TAT). The preoperative plasma TAT was dichotomised into high (≥3.2 ng/mL) and low (<3.2 ng/mL). Association between plasma TAT and overall survival is shown. Univariate statistical testing was performed using Cox proportional hazards model (multivariate testing is detailed in Appendix D). Multivariate p‐value and hazard ratio shown

Pre‐operative plasma TAT (*P* = .02) and D‐dimer (*P* < .05) were associated with reduced OS. On multivariate analysis, pre‐operative plasma TAT was associated with reduced OS (HR 3.26, CI:1.16‐3.1, *P* = .02) with a high plasma TAT (≥3.2 ng/mL) associated with greater than three‐fold mortality risk compared to low plasma TAT (<3.2 ng/mL) (Figure 5, Appendix D).

Plasma TF, TAT, and D‐dimer did not correlate with epithelial or fibroblast expression of TF, thrombin, PAR1, or PAR2, unlike a previous small study.^17^

## 4 DISCUSSION

This large prospective study investigates the stromal, epithelial, and systemic activation of the extrinsic clotting pathway in invasive breast cancer, compared to DCIS and normal breast tissue. We demonstrate that a procoagulant fibroblast phenotype is associated with more aggressive breast cancer subtypes and reduced survival, with this procoagulant fibroblast phenotype developing at the preinvasive stage.

### 4.1 Procoagulant fibroblasts in DCIS: Facilitators or biomarkers of invasion

In this cohort, extrinsic clotting pathway expression (TF, thrombin and their receptors PAR2 and PAR1) is increased in stromal fibroblasts in both DCIS and invasive cancer compared to normal breast tissue. This implies paracrine interactions between the malignant epithelium, within the duct basement membrane, and the surrounding stroma at the preinvasive stage. TF‐expressing myoepithelial cells have previously been reported in normal breast tissue and around nests of invasive cells in comedo DCIS.^18^ However, stromal fibroblasts, and not myoepithelial cells, were the focus of this current study. We demonstrate a procoagulant fibroblast phenotype diffusely throughout the stroma (and not just in the periductal tissue) of DCIS.

CAFs, the most abundant cell of the tumor microenvironment, are phenotypically and functionally different from normal fibroblasts and promote tumor growth and invasion. In vivo co‐injection of premalignant breast cells and normal fibroblasts form DCIS; however, co‐injection with CAFs results in invasive tumors and metastases.^3^, ^7^ Breast CAFs have a distinctive gene expression profile compared to normal breast fibroblasts.^19^ In DCIS, stromal gene expression profiles mirror the respective invasive signatures, highlighting that gene expression changes occur largely at the preinvasive stage,^20^ and this is reflected here with a procoagulant phenotype in DCIS fibroblasts, similar to CAFs in invasive breast cancer. This development of a procoagulant phenotype in fibroblast transitioning to CAF is supported by α‐SMA expressing fibroblasts (CAFs) but not quiescent fibroblasts expressing PAR1 and PAR2 in vitro.^21^ Whether this procoagulant CAF phenotype is the result of paracrine stimulation from malignant epithelial cells or is a primary stromal environmental change is unclear; however, recent preclinical data report that more aggressive breast cancer cell lines induce a CAF phenotype via Wnt7a.^22^ Two translational studies have demonstrated evidence of upregulated procoagulant tumor phenotype in relation to breast cancer stroma. Increased stromal gene expression of the thrombin receptor PAR3 was associated with early recurrence in breast cancer.^6^ In triple negative cancers, genes enriched in stroma compartment (compared to the Cancer Genome Atlas) included upregulation of genes for PAR1 as well as Coagulation Factor XIIIa which is activated (from XIII) by thrombin.^23^

In the current study, the procoagulant transition of CAFs is only mirrored by thrombin, but not PAR1, PAR2, and TF epithelial over‐expression, suggesting that stromal, not epithelial, procoagulant changes are drivers of cancer behavior. Clotting factors may directly facilitate destruction of the myoepithelial layer and progression to invasion or may create a “wound that does not heal.”^8^ The latter is supported the by coevolution of the immune microenvironment with breast cancer progression with for example, a reduction in activated CD8^+^ and TIGIT^+^ T cells and T‐cell receptor clonal diversity, but an increase in PD‐L1 expression with transition from DCIS to invasion.^24^

### 4.2 Procoagulant fibroblasts in breast cancer: Functional promoters or biomarkers of poor outcome?

Although breast CAFs are morphologically homogeneous, gene expression profiles vary between invasive breast cancer subtypes.^25^ Stromal genetic profiling predicts prognosis independent of ER, HER2, grade, nodal status, and previous treatments.^6^ For example, fibroblasts with a “wound‐healing” genotype are predictors of metastases and death in breast cancer.^26^ Different CAF phenotypes are heterogeneous in their tumor‐promoting properties with, for example, HGF‐ and HSF1‐expressing,^27^, ^28^ and Caveolin‐1‐deficient^29^ fibroblasts all demonstrating increased tumor‐inducing ability. Stromal phenotype differs according to breast cancer subtype.^9^, ^30^ Here, a more procoagulant fibroblast phenotype occurred in high grade, high proliferation, ER‐negative, and HER2‐positive breast cancers, features associated with poor outcome. Similarly we found a procoagulant fibroblast phenotype associated with ER‐negative DCIS, a subtype at increased risk of recurrence.^31^ A procoagulant fibroblast phenotype was not associated with tumor size or node positivity, implying that stromal procoagulant changes are a function of tumor biology and not a time‐dependent feature. The procoagulant expression in poor prognosis disease could represent a non‐functional bystander effect, with the potential to have utility as a prognostic biomarker. However, in vitro evidence supports a functional role for coagulation in the development of CAFs. Thrombin‐PAR1 activation of fibroblasts induces a synchronous CAF‐like (α‐SMA) and procoagulant (TF) fibroblast phenotype.^32^, ^33^ In addition, TGF‐β induced transformation of normal fibroblasts to CAF stimulates TF expression.^18^, ^34^

In CHAMPion, D‐dimer was an independent predictor of lymph node metastasis, confirming two smaller studies.^35^, ^36^ Downstream extrinsic clotting pathway activation, including the formation of fibrin, is required for the successful establishment of metastases.^14^ Fibrin, is degraded into D‐dimer by the plasminogen activator system, an enzymatic cascade that is important in the degradation of extracellular matrix proteins required for invasion into the lymphovascular space.^37^ The raised D‐dimer in patients with lymph node metastasis may reflect systemic or tumor clotting activation and fibrinolysis in the presence of lymphovascular invasion.

There are some limitations to our study. First, TMA (vs whole slide) analyses pose a challenge where intratumoral heterogeneity exists. However, in a study of over 1000 breast cancer cases, heterogeneity as a source of discordance between whole slide and 1.0 mm TMA was between 2% and 8% for ER and PR (which shows nuclear expression) and HER2 (which shows cytoplasmic). Overall, agreement between TMA and whole slide scoring was high for ER (94%), PR (89%), and HER2 (88%) for manually scored TMAs.^38^ Another similarly sized study of multiple cores taken from representative areas showed that 81% of cores were suitable for analysis with a 99% concordance with the whole sections for the cytoplasmic marker HER2.^39^ Increased concordance is seen with 1.0 vs 0.6 mm and for multiple cores versus just one (we have done two cores of 1.0 mm per whole slide).^40^, ^41^

Fibroblasts were identified by morphology, rather than co‐staining with a fibroblast or CAF marker. However, fibroblasts have a distinct morphology and were defined and co‐scored by experienced histopathologists. The normal breast control tissue was an intra‐patient control, with histopathologically determined normal tissue taken at a distance from DCIS and invasive breast cancers. This allowed for precise demographic matching of the cancer and control group. In addition, gene expression profiles of morphologically normal breast tissue adjacent to cancers are no different to that of breast reduction tissue.^42^

### 4.3 Procoagulant CAFs as a novel therapeutic target

CAFs are an emerging therapeutic target in cancer as they may have a role in treatment resistance. Extrinsic clotting pathway inhibitors are effective anti‐cancer therapies in in vivo murine studies, with the anti‐human TF antibody CNTO‐859 and several thrombin inhibitors including Dabigatran (a direct oral anticoagulant in wide clinical use for thromboprophylaxis) inhibiting tumor initiation, growth, and metastases.^43^, ^44^, ^45^ Dabigatran also inhibits thrombin‐induced fibroblast activation in vitro.^32^

CHAMPion highlights that procoagulant fibroblasts are most evident in ER‐negative cancer, a subgroup with more limited treatment options. Targeting procoagulant CAFs as an adjunct to conventional therapies may have efficacy in all breast cancer patients, but particularly this challenging, poor‐prognosis subgroup. This is currently being investigated in a phase II randomised controlled window‐of‐opportunity trial of Rivaroxaban in ER‐negative early breast cancer patients (ISRCTN14785273).^46^

This study provides evidence for an epithelial‐stromal communication across the basement membrane in DCIS, creating a procoagulant stromal milieu. A procoagulant phenotype in invasive cancer correlates with aggressive breast cancer subtypes and reduced survival, highlighting the potential of the clotting pathway as a prognostic biomarker. Of greater clinical significance, this study provides evidence that coagulation may facilitate breast cancer progression. The clotting pathway is a potential novel therapeutic target that may have the potential to prevent invasion in patients diagnosed with DCIS. In addition, the clotting pathway may have potential as a target to inhibit metastases and be used as an adjunct to established breast cancer treatment, particularly in ER‐negative, HER2‐positive disease.

## CONFLICT OF INTEREST STATEMENT

The authors declare no potential conflicts of interest.

## AUTHORS' CONTRIBUTIONS

CCK, NJB, and GL conceptualized the study. CCK, HS, NJB, GL, JC, and SAP developed the methodology. CCK, HS, JC, GL, HA, SLN, LJH, JYEH, and SAP performed data curation and formal analysis. CCK, HS, and JC performed project administration. CCK supervised the study. CCK and NJB performed funding acquisition. HS and CCK wrote the original draft and all authors contributed to reviewing and editing the final draft.
