# Supplementary material for: Breast cancer stromal clotting activation (Tissue Factor and thrombin): A pre‐invasive phenomena that is prognostic in invasion
Source: Cancer Med. 2020 Jan 21;9(5):1768–78. doi: 10.1002/cam4.2748 (PMC7050075; doi:10.1002/cam4.2748)
Supplement: Supplementary file 1 [file CAM4-9-1768-s001.docx]

**Appendix A**

1. STROBE checklist page 1

i) Table A. Checklist of items that should be included in reports of cohort studies page 1

ii) Checklist points addressed for CHAMPion cohort study page 3

i) Figure A: STROBE diagram page 6

1. REMARK checklist page 7

## STROBE checklist for CHAMPion cohort study

## Strobe checklist is shown below with specific items for the CHAMPion cohort study addressed below it. *i) Table A. Checklist of items that should be included in reports of cohort studies*

|  | Item No | Recommendation |
| --- | --- | --- |
| Title and abstract | 1 | (*a*) Indicate the study’s design with a commonly used term in the title or the abstract |
|  |  | (*b*) Provide in the abstract an informative and balanced summary of what was done and what was found |
| Introduction | | |
| Background/rationale | 2 | Explain the scientific background and rationale for the investigation being reported |
| Objectives | 3 | State specific objectives, including any prespecified hypotheses |
| Methods | | |
| Study design | 4 | Present key elements of study design early in the paper |
| Setting | 5 | Describe the setting, locations, and relevant dates, including periods of recruitment, exposure, follow-up, and data collection |
| Participants | 6 | (*a*) Give the eligibility criteria, and the sources and methods of selection of participants. Describe methods of follow-up |
|  |  | (*b*) For matched studies, give matching criteria and number of exposed and unexposed |
| Variables | 7 | Clearly define all outcomes, exposures, predictors, potential confounders, and effect modifiers. Give diagnostic criteria, if applicable |
| Data sources/ measurement | 8* | For each variable of interest, give sources of data and details of methods of assessment (measurement). Describe comparability of assessment methods if there is more than one group |
| Bias | 9 | Describe any efforts to address potential sources of bias |
| Study size | 10 | Explain how the study size was arrived at |
| Quantitative variables | 11 | Explain how quantitative variables were handled in the analyses. If applicable, describe which groupings were chosen and why |
| Statistical methods | 12 | (*a*) Describe all statistical methods, including those used to control for confounding |
|  |  | (*b*) Describe any methods used to examine subgroups and interactions |
|  |  | (*c*) Explain how missing data were addressed |
|  |  | (*d*) If applicable, explain how loss to follow-up was addressed |
|  |  | (*e*) Describe any sensitivity analyses |
| Results | | |
| Participants | 13* | (a) Report numbers of individuals at each stage of study—eg numbers potentially eligible, examined for eligibility, confirmed eligible, included in the study, completing follow-up, and analysed |
|  |  | (b) Give reasons for non-participation at each stage |
|  |  | (c) Consider use of a flow diagram |
| Descriptive data | 14* | (a) Give characteristics of study participants (eg demographic, clinical, social) and information on exposures and potential confounders |
|  |  | (b) Indicate number of participants with missing data for each variable of interest |
|  |  | (c) Summarise follow-up time (eg, average and total amount) |
| Outcome data | 15* | Report numbers of outcome events or summary measures over time |
| Main results | 16 | (*a*) Give unadjusted estimates and, if applicable, confounder-adjusted estimates and their precision (eg, 95% confidence interval). Make clear which confounders were adjusted for and why they were included |
|  |  | (*b*) Report category boundaries when continuous variables were categorized |
|  |  | (*c*) If relevant, consider translating estimates of relative risk into absolute risk for a meaningful time period |
| Other analyses | 17 | Report other analyses done—eg analyses of subgroups and interactions, and sensitivity analyses |
| Discussion | | |
| Key results | 18 | Summarise key results with reference to study objectives |
| Limitations | 19 | Discuss limitations of the study, taking into account sources of potential bias or imprecision. Discuss both direction and magnitude of any potential bias |
| Interpretation | 20 | Give a cautious overall interpretation of results considering objectives, limitations, multiplicity of analyses, results from similar studies, and other relevant evidence |
| Generalisability | 21 | Discuss the generalisability (external validity) of the study results |
| Other information | | |
| Funding | 22 | Give the source of funding and the role of the funders for the present study and, if applicable, for the original study on which the present article is based |

***ii) Checklist points addressed for CHAMPion cohort study***

1. **Title and abstract**
   1. Title does not refer to cohort study but is clear from abstract
   2. Cohort study mentioned in abstract
2. **Introduction**Background/rationale: Explained in introduction
3. **Introduction**
   Objectives: Explained in introduction and methods
4. **Methods**
   Study design: Design of prospective cohort (CHAMPion study) presented clearly in methods
5. **Methods**
   Setting: Recruitment location, recruitment dates .and data collection detailed in methods. No follow up of prospective cohort study described here.
6. **Participants**
   1. Eligibility and exclusion criteria provided in methods.
   2. N/A
7. **Variables**Exclusion criteria includes variables that may act as potential confounders. Potential confounders and effect modifiers discussed with regards to assessmet of peri-operative plasma markers.
8. **Data sources/measurement**Detailed in methodology and supplementary data.
9. **Bias**No clear causes of bias identified in cohort study design.
10. **Study size**Power calculation for prospective cohort study provided.
11. **Quantitative variables**Summarised in statistical methods section.
12. **Statistical methods**
    1. All statistical methods described
    2. N/A (subgroup analyses)
    3. Missing data: CHAMPion cohort is not followed up. No statistical adjustments made for missing data.
    4. General Practitioners contacted
    5. N/A (sensitivity analyses)
13. **Participants**
    1. Number of individuals at each stage of study reported in results
    2. Reasons given
    3. Flow diagram inserted (Figure 2)
14. **Descriptive data**
    1. Characteristics of study participants provided in Table 1
    2. Numbers and percentages provided for all variables
    3. Provided in results
15. **Outcome data**Reported in results
16. **Main results**
    1. Results given with precision estimates (Standard error or 95% confidence interval where relevant)
    2. Category boundary: Reported
    3. Hazard ratio explained in more clinically relevant way
17. **Other analyses**No other analyses done.
18. **Key results (Discussion)**Reported
19. **Limitations**Stated
20. **Interpretation**Stated
21. **Generalisability**Stated **OTHER INFORMATION**
22. **Funding**
    Stated


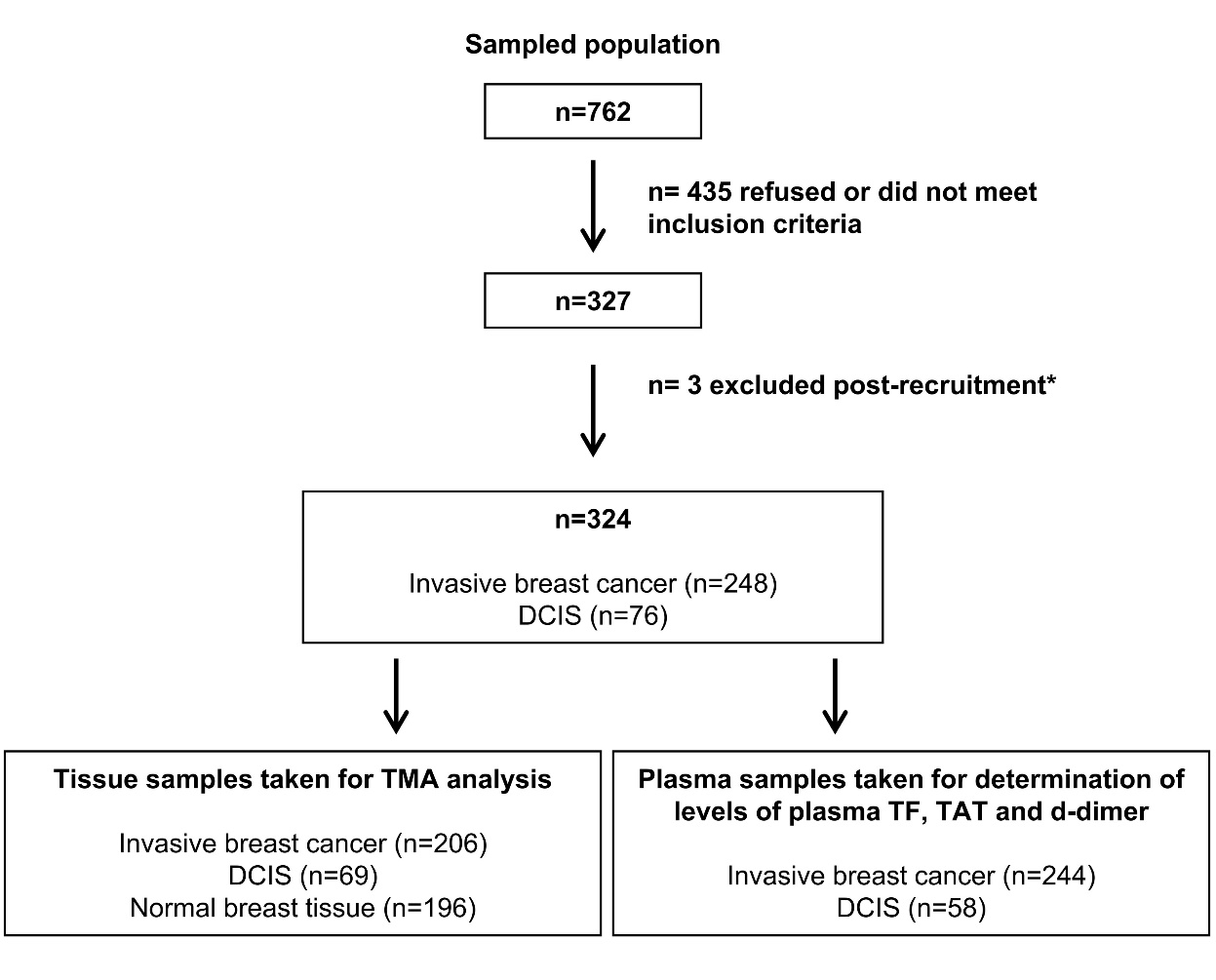


**Figure 1: STROBE diagram for CHAMPion study patient recruitment in the CHAMPion study**Exclusion criteria: previous neoadjuvant chemotherapy; history of VTE or thrombophilia; current anticoagulant therapy; severe immobility. *One patient excluded due to family history of Factor V Leiden coagulopathy and two due to post-recruitment identification of history venous thromboembolism.

**REMARK checklist for CHAMPion cohort study**

As per J Clin Oncol. 2005 Dec 20;23(36):9067-72. Reporting recommendations for tumor marker prognostic studies. McShane LM, Altman DG, Sauerbrei W, Taube SE, Gion M, Clark GM; Statistics Subcommittee of the NCI-EORTC Working Group on Cancer Diagnostics.

**Introduction:**

Marker, objectives, hypotheses stated, page 5

**Methods**

Patients: Described page 5, Fig.2

Specimen: page 5

Assay methods: pages 5-7, S1,2

Study Design: pages 5-8, S5

Statistical Analysis: pages 7,8,11, S5

**Results**

Data: Fig. 2, Table 1

Analysis and Presentation Figs 2-7, pages 8-11, S3-7

**Discussion**

Pages 12-17
