# Supplementary material for: Breast cancer stromal clotting activation (Tissue Factor and thrombin): A pre‐invasive phenomena that is prognostic in invasion
Source: Cancer Med. 2020 Jan 21;9(5):1768–78. doi: 10.1002/cam4.2748 (PMC7050075; doi:10.1002/cam4.2748)
Supplement: Supplementary file 2 [file CAM4-9-1768-s002.docx]

**Appendix B: Details of cell culture and antibody validation for immunohistochemistry**

**Cell lines**

Human breast cancer cell lines MDAMB231, MDAMB468, MCF7, T47D and SKBR3 used throughout this study were purchased from the American Type Culture Collection (ATCC).

Summary of the origin and receptor status of breast cancer cell lines

| **Cell line** | **Description** | **ER/PR/HER2 status** |
| --- | --- | --- |
| MDAMB-231 | Pleural effusion derived adenocarcinoma cell line | ER negative, PR negative, HER2 normal/low |
| MDAMB-468 | Pleural effusion derived adenocarcinoma cell line | ER negative, PR negative, HER2 normal/low |
| MCF-7 | Pleural effusion derived adenocarcinoma cell line | ER positive, PR positive, HER2 normal/low |
| T47D | Pleural effusion derived adenocarcinoma cell line | ER positive, PR positive, HER2 normal/low |
| SKBR3 | Pleural effusion derived adenocarcinoma cell line | ER negative, PR negative, HER2 overexpressing |
| **ER** oestrogen receptor **PR** progesterone receptor **HER2** Human epidermal growth factor receptor 2 | | |

**Antibodies**

| **Antibody** | **Species** | **Company** | **Cat No.** | **Application (dilution, incubation time, temperature)** |
| --- | --- | --- | --- | --- |
| Tissue Factor | Mouse | Axis Shield | ADI4508 | WB (1:250, O/N, 4^o^C) IHC (1:50, 1hr, RT) |
| β actin | Goat | Santa Cruz | SC-1616 | WB (1:500, 1hr, RT) |
| Goat IgG conjugated to HRP | Rabbit | Dako | P0449 | WB (1:500, 1hr, RT) |
| Mouse IgG conjugated to HRP | Sheep | GE Healthcare | NA931V | WB (1:5000, 1hr, RT) |
| Thrombin | Rabbit | abcam | Ab83981 | WB (1:200, O/N, 4^o^C) IHC (1:1000, 1hr, RT) |
| PAR1 | Mouse | Santa Cruz | Sc-13503 | WB (1:50, O/N, 4^o^C) IHC (1:100, 1hr, RT) |
| PAR2 | Mouse | Santa Cruz | SC-13504 | WB (1:250, O/N, 4^o^C) IHC (1:50, 1hr, RT) |
| **O/N** overnight **RT** room temperature **WB** Western blot  **IHC** immunohistochemistry **FACS** Fluorescence assisted cell sorting **HRP** horseradish peroxidase | | | | |

**Cell culture**

Human breast cancer cell lines were maintained in adherent culture conditions at 37^o^C at atmospheric pressure in 5% (v/v) carbon dioxide/air (Humidified Sanyo incubator). These conditions were used for all experiments. MCF-7, T47D and SKBR3 cells lines were cultured in complete DMEM medium while MDAMB-231 and MDAMB-468 cell lines were cultured in complete RPMI-1640 medium. Cells were passaged when approximately 80% confluent. Cell lines were authenticated by multiplex PCR assay using the AmpF/STR system (Applied Biosystems) and confirmed as mycoplasma free on a regular basis. Cell lines were not cultured beyond 20 passages.

**Testing antibody specificity on breast cancer cell lines by western blotting and immunohistochemistry**

Antibodies used were mouse anti-human TF (ADG4508, American Diagnostica, Sekisui Diagnostics, Lexington, MA), rabbit anti-human thrombin (ab83981, Abcam, Cambridge, UK), mouse anti-human PAR1 (sc13503, Santa Cruz Biotech, California) and mouse anti-human PAR2 (sc13504, Santa Cruz Biotech, California). Antibodies against TF, PAR1 and PAR2 have been previously been published in several studies including in breast cancer. To test antibody specificity, a comparison was made between protein detection via western blotting (using a cell lysate) and via immunohistochemistry (using a cell pellet) on a panel of breast cancer cell lines. Two ER positive cell lines (MCF-7, T47D) and three ER-negative cell lines (MDA-MB-231, MDA-MB-468 and SKBR3) were used. Semi-confluent cells, maintained in adherent culture as previously described from the five cell lines, were trypsinised and centrifuged to create two cell pellets. One pellet was lysed using standard protein lysis buffer and stored at -20^o^C until protein expression was determined by western blotting. The other pellet was fixed overnight in 4% formalin. Cells were then washed in PBS, spun down and resuspended in molten (<50^o^C) agarose and allowed to set at 2-8^o^C in the Histology department at the Cancer Research UK Manchester Institute. 4µm sections were created from paraffin embedded cells and protein expression was determined on cells from the same passage using immunohistochemistry as detailed below.

**Immunohistochemistry**

Sections from TMA blocks or cell pellet blocks were prepared and stained at the Centre for Molecular Pathology at Lund University, Malmo by a single histology technician. Sections were deparaffinised and antigen retrieval performed using an automated tissue pre-treatment module PT Link (Dako, Cambridgeshire, UK) with EnVision™ FLEX Target Retrieval Solution (K8004, Dako) at pH 9 for 20 minutes at 97^o^C. Immunohistochemistry was performed with Dako’s s Autostainer Plus with EnVision™ FLEX High pH (K8010, Dako) at room temperature for one hour using 1:50 anti-TF antibody, 1:1000 anti-Thrombin antibody, 1:100 PAR1 antibody and 1:50 antibody. All sections were counterstained with haematoxylin. Antibody concentrations were decided based on manufacturer’s recommendations as well as being optimised at Lund University to minimise background and maximise specificity.

**Measurement of plasma extrinsic clotting pathway markers**

A 20ml sample of venous blood was collected using evacuated tube systems into buffered citrate collection tubes prior to surgical resection. Three markers of systemic (plasma) thrombin pathway activation were measured; TF, thrombin-antithrombin (TAT) and D-dimer. Plasma D-dimer measurement was performed on fresh citrated samples by the Anticoagulation and Haematology Research Unit (AHRU) at University Hospital of South Manchester. From the start of patient recruitment (August 2010) until December 2012 plasma d-dimer measurement was performed using miniVIDAS® (bioMérieux Clinical Diagnostics, Marcy L’Etoile, France). From December 2012 onwards, the AHRU measured D-dimer using ACL-TOP500 (Instrumentation Laboratory, Bedord, MA, USA). The two techniques have been shown to be comparable in their detection of d-dimer in previous publications 20 and were also validated internally. Remaining citrated blood was centrifuged at 3500g for 20 minutes at 4^o^C. Plasma was stored at -80˚C until assayed for TF using the Imubind® ELISA kit (Sekuisi, MA) and for TAT using the TAT Micro Kit (Sysmex, UK). Completed ELISA plates were read using FLUOstar Omega (BMG Labtech, Ortenberg, Germany) and values calculated using 4-parameter logistic regression analysis. Half the detection limit value for each ELISA kit was used for statistical analysis in case the measured values did not reach the detection limit of the assay.
