# Supplementary material for: Breast cancer stromal clotting activation (Tissue Factor and thrombin): A pre‐invasive phenomena that is prognostic in invasion
Source: Cancer Med. 2020 Jan 21;9(5):1768–78. doi: 10.1002/cam4.2748 (PMC7050075; doi:10.1002/cam4.2748)
Supplement: Supplementary file 3 [file CAM4-9-1768-s003.docx]

## Appendix C:

## TMA scoring of epithelial expression of Tissue Factor, PAR1 and PAR2 (Thrombin epithelial scoring detailed in Figure 1)

Figure C.1. TMA scoring of epithelial TF expression page 2

Figure C.2. TMA scoring of epithelial PAR1 expression page 3

Figure C.3. TMA scoring of epithelial PAR2 expression page 4


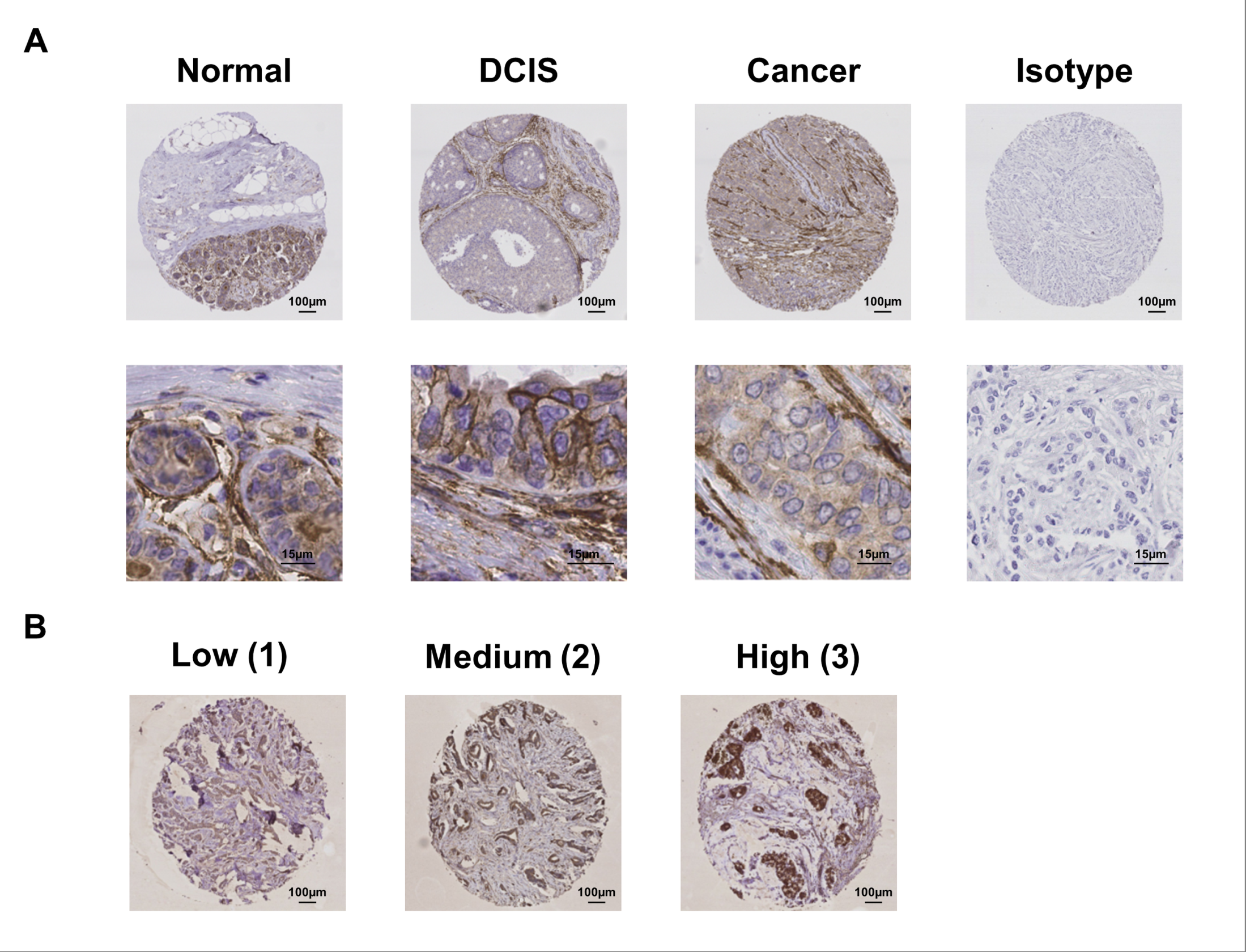


**Figure C.1. TMA scoring of epithelial TF expression**(A) Immunohistochemical staining for TF in breast TMA cores, showing examples of invasive cancer, DCIS and normal breast tissue as well as cancer tissue stained with an antibody isotype control. (B) Immunohistochemical staining of TF showing epithelial scoring at levels 1 (low), 2 (medium) and 3 (high) at x6 magnification. Mean score was used to dichotomise scores into high and low.


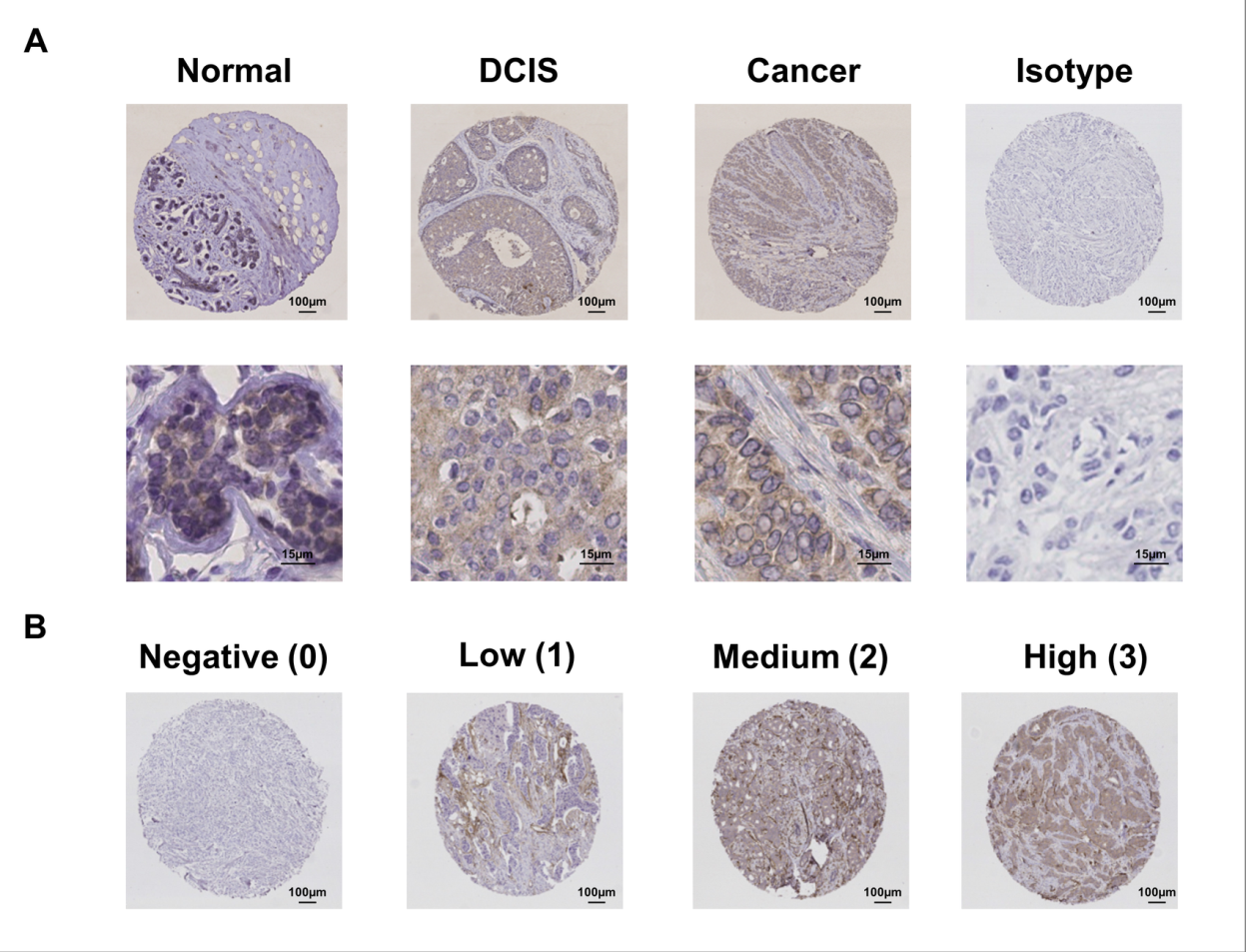


**Figure C.2. TMA scoring of epithelial PAR1 expression**(A) Immunohistochemical staining for PAR1 in breast TMA cores, showing examples of invasive cancer, DCIS and normal breast tissue as well as cancer tissue stained with an antibody isotype control. (B) Immunohistochemical staining of PAR1 showing epithelial scoring at levels 1 (low), 2 (medium) and 3 (high) at x6 magnification.


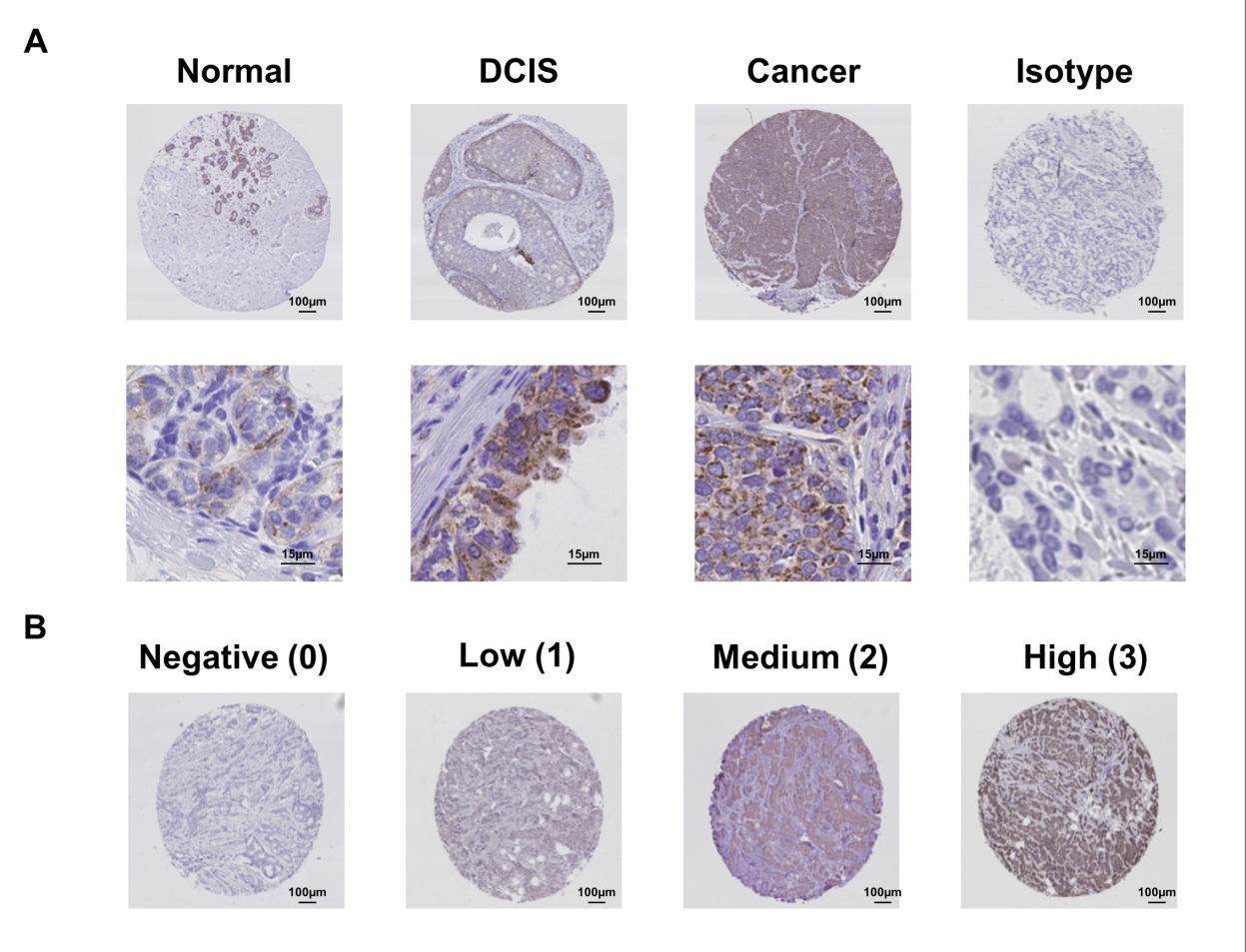


**Figure C.3. TMA scoring of epithelial PAR2 expression**(A) Immunohistochemical staining for PAR2 in breast TMA cores, showing examples of invasive cancer, DCIS and normal breast tissue as well as cancer tissue stained with an antibody isotype control. (B) Immunohistochemical staining of PAR2 showing epithelial scoring at levels 1 (low), 2 (medium) and 3 (high) at x6 magnification.
