# Supplementary material for: Breast cancer stromal clotting activation (Tissue Factor and thrombin): A pre‐invasive phenomena that is prognostic in invasion
Source: Cancer Med. 2020 Jan 21;9(5):1768–78. doi: 10.1002/cam4.2748 (PMC7050075; doi:10.1002/cam4.2748)
Supplement: Supplementary file 4 [file CAM4-9-1768-s004.docx]

**Appendix D:** **Details of analysis for overall and disease-free survival in invasive cancer patients**

**Overall survival data for invasive cancer patients**

1. **Details of univariate and analysis for overall survival**

| **Variable** | | **Hazard ratio** | **95% CI** | **p** |
| --- | --- | --- | --- | --- |
| **Univariate analysis** | |  |  |  |
|  | HER2 status  (negative vs positive) | 1.21 | 0.27-5.35 | 0.8 |
|  | Lymph node status (negative vs positive) | 2.87 | 1.04-7.93 | 0.04* |
|  | ER status  (negative vs positive) | 0.225 | 0.082-0.62 | 0.004* |
|  | Tumour size | 1.06 | 1.03-1.08 | 0.001* |
|  | Grade  (grade 1 vs grade 2 and grade 3) |  |  | 0.007* (overall) |
|  | Grade 2    Grade 3 | 0.64  4.8 | 0.08-4.41  1.07-21.3 | 0.63  0.41 |
|  | Ki67 expression  (<20% vs >20%) | 1.038 | 1.02-1.06 | 0.0001 |
|  | Age | 1.02 | 0.97-1.08 | 0.458 |
|  | TF fibroblast expression | 1.05 | 1.007 – 1.09 | 0.02 |
|  | Thrombin fibroblast expression | 1.02 | 0.99-1.04 | 0.21 |
|  | PAR1 fibroblast expression | 1.2 | 0.42-3.73 | 0.68 |
|  | PAR2 fibroblast expression | 1.06 | 0.34-3.29 | 0.91 |
|  | TF epithelial expression  (low vs high) | 5.33 | 1.62-17.49 | 0.006* |
|  | Thrombin epithelial expression  (low vs high) | 1.26 | 0.41-3.93 | 0.69 |
|  | PAR1 epithelial expression  (low vs high) | 1.31 | 0.42-4.15 | 0.64 |
|  | PAR2 epithelial expression (low vs high) | 0.91 | 0.29-2.87 | 0.87 |
|  | Pre-operative plasma TF (low vs high) | 1.39 | 0.5-3.8 | 0.53 |
|  | Pre-operative plasma TAT (low vs high) | 3.3 | 1.2-9.1 | 0.021* |
|  | Pre-operative plasma D-dimer (low vs high) | 2.86 | 1.02-8.02 | 0.047* |

** Predictors of overall survival on univariate analysis at p<0.1; entered into multivariate cox proportional hazards model*

Steps in backward step-wise selection model for clinicopathological variables

Backward step-wise selection was used to select significant variables to enter into a cox proportional hazard model with tissue and plasma markers that were significant on univariate analysis.

Step 1

| **Variable** | **P value** |
| --- | --- |
| Age | 0.056 |
| Ki67 | 0.38 |
| Tumour size | <0.001 |
| ER status | 0.748 |
| Invasive Grade | 0.118 |
| Nodal status | 0.719 |

Step 2

| **Variable** | **P value** |
| --- | --- |
| Age | 0.044 |
| Ki67 | 0.191 |
| Tumour size | <0.001 |
| Invasive Grade | 0.118 |
| Nodal status | 0.752 |

Step 3

| **Variable** | **P value** |
| --- | --- |
| Age | 0.043 |
| Ki67 | 0.167 |
| Tumour size | <0.001 |
| Invasive Grade | 0.123 |

Step 4

| **Variable** | **P value** |
| --- | --- |
| Age | 0.032 |
| Tumour size | <0.001 |
| Invasive Grade | 0.005 |

**Multivariate analysis of factors entered into cox proportional hazards model for overall survival after backward step-wise selection**

| **Variable** | | **Hazard ratio** | **95% CI** | **p** |
| --- | --- | --- | --- | --- |
| **Clinicopathological variables** | |  |  |  |
|  | Age | 1.039 | 0.98-1.09 | 0.17 |
|  | Tumour size | 1.04 | 1.01-1.07 | 0.008 |
|  | Invasive Grade |  |  | 0.055 |
| **Tissue/Plasma markers** | |  |  |  |
|  | Pre-operative plasma TAT  (High vs Low) | 3.25 | 1.16-9.12 | 0.025 |
|  | Pre-operative plasma D-dimer (High vs Low) | 1.34 | 0.40-4.74 | 0.61 |
|  | TF epithelial expression | 10.62 | 2.98-37.7 | 0.0001 |
|  | TF fibroblast expression | 1.038 | 0.994-1.084 | 0.092 |

**Disease-free and overall survival data for invasive breast cancer patients**

1. **Details of univariate and analysis for overall survival**

| **Variable** | | **Hazard ratio** | **95% CI** | **p** |
| --- | --- | --- | --- | --- |
| **Univariate analysis** | |  |  |  |
|  | HER2 status  (negative vs positive) | 0.81 | 0.18-3.68 | 0.78 |
|  | Lymph node status (negative vs positive) | 3.98 | 1.54-10.3 | 0.004* |
|  | ER status  (negative vs positive) | 0.287 | 0.11-0.75 | 0.011* |
|  | Tumour size | 1.059 | 1.04-1.08 | <0.001* |
|  | Grade  (grade 1 vs grade 2 and grade 3) |  |  | 0.004* (overall) |
|  | Grade 2    Grade 3 | 0.879  5.35 | 0.15-1.2  1.20-2.39 | 0.88  0.028 |
|  | Ki67 expression  (<20% vs >20%) | 3.27 | 1.16-9.2 | 0.025* |
|  | Age | 1.023 | 0.97-1.07 | 0.37 |
|  | TF fibroblast expression | 1.035 | 1.001 – 1.07 | 0.041* |
|  | Thrombin fibroblast expression | 1.02 | 0.98-1.02 | 0.86 |
|  | PAR1 fibroblast expression | 1.003 | 0.98-1.02 | 0.76 |
|  | PAR2 fibroblast expression | 0.99 | 0.971-1.02 | 0.68 |
|  | TF epithelial expression  (low vs high) | 4.7 | 1.5-14.6 | 0.007* |
|  | Thrombin epithelial expression  (low vs high) | 1.02 | 0.34-3.03 | 0.97 |
|  | PAR1 epithelial expression  (low vs high) | 1.14 | 0.37-3.49 | 0.81 |
|  | PAR2 epithelial expression (low vs high) | 0.81 | 0.26-2.47 | 0.71 |
|  | Pre-operative plasma TF (low vs high) | 0.696 | 0.24-2.0 | 0.50 |
|  | Pre-operative plasma TAT (low vs high) | 2.65 | 0.94-7.44 | 0.065* |
|  | Pre-operative plasma D-dimer (low vs high) | 3.19 | 0.89-0.247 | 0.86 |

Steps in backward step-wise selection model for clinicopathological variables
Backward step-wise selection was used to select significant variables to enter into a cox proportional hazard model with tissue and plasma markers that were significant on univariate analysis.

Step 1

| **Variable** | **P value** |
| --- | --- |
| Age | 0.047 |
| Ki67 | 0.4 |
| Tumour size | <0.001 |
| ER status | 0.779 |
| Invasive Grade | 0.07 |
| Nodal status | 0.45 |

Step 2

| **Variable** | **P value** |
| --- | --- |
| Age | 0.048 |
| Ki67 | 0.403 |
| Tumour size | <0.001 |
| Invasive Grade | 0.073 |
| Nodal status | 0.446 |

Step 3

| **Variable** | **P value** |
| --- | --- |
| Age | 0.049 |
| Ki67 | 0.427 |
| Tumour size | <0.001 |
| Invasive Grade | 0.06 |

Step 4

| **Variable** | **P value** |
| --- | --- |
| Age | 0.041 |
| Tumour size | <0.001 |
| Invasive Grade | 0.002 |

**Multivariate analysis of factors entered into cox proportional hazards model for disease-free survival after backward step-wise selection**

| **Variable** | | **Hazard ratio** | **95% CI** | **p** |
| --- | --- | --- | --- | --- |
| **Clinicopathological variables** | |  |  |  |
|  | Age | 1.01 | 0.98-1.045 | 0.55 |
|  | Tumour size | 1.05 | 1.03-1.08 | <0.001 |
|  | Invasive Grade |  |  | 0.036 |
| **Tissue/Plasma markers** | |  |  |  |
|  | Pre-operative plasma TAT  (High vs Low) | 2.5 | 0.93-6.68 | 0.067 |
|  | TF epithelial expression (Low vs High) | 19.4 | 4.9-75.4 | <0.001 |
|  | TF fibroblast expression | 1.02 | 0.98-1.05 | 0.31 |


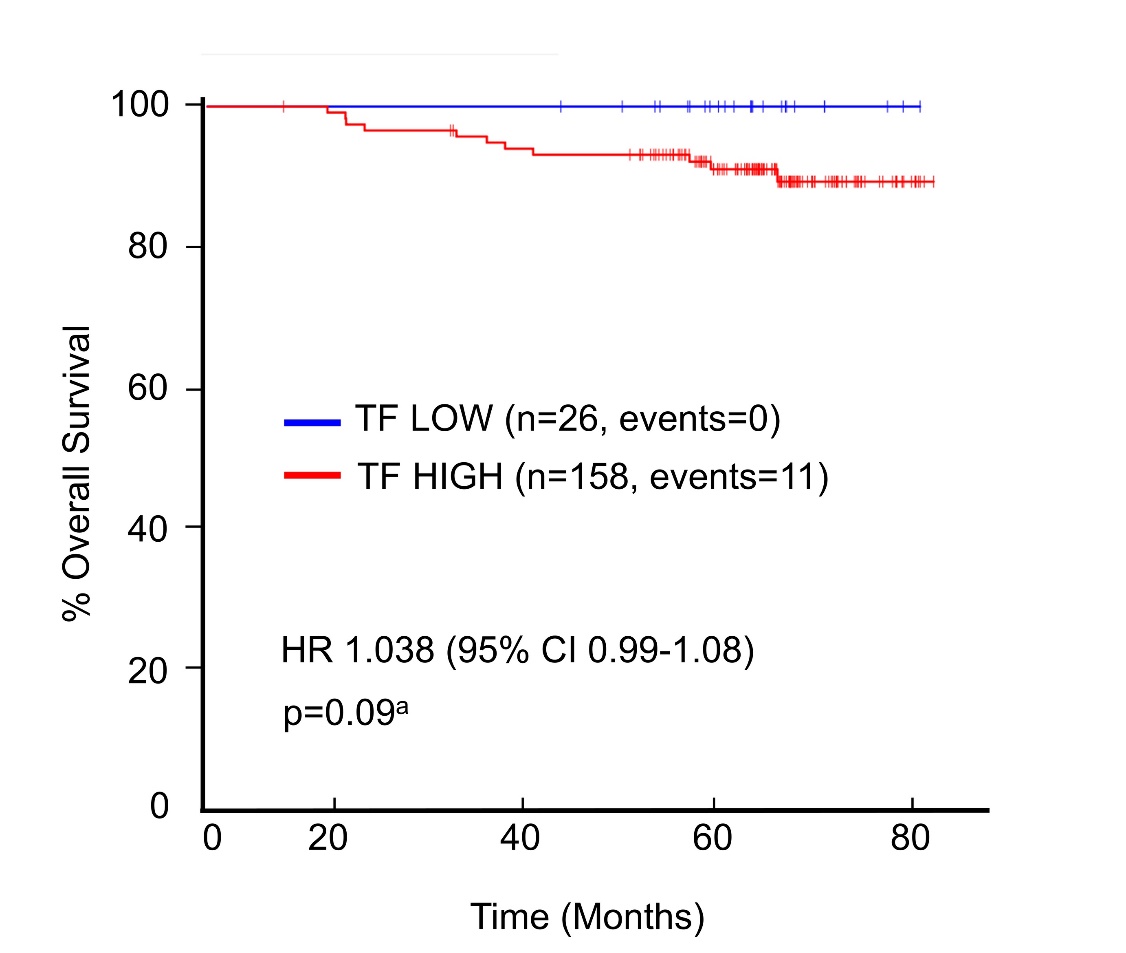


**Figure D.1: Kaplan-Meier survival curves for overall survival according to TF fibroblast expression**The percentage of stromal fibroblasts expressing TF was estimated and dichotomised into high (50%) and low (≤50%) expression. Association between fibroblast expression of TF and DFS is shown. Univariate statistical testing was performed using Cox proportional hazards model (multivariate testing is detailed in Appendix D). TF-low: ≤50% fibroblast expression, TF-high: >50% fibroblast expression.

^a^Multivariate p-value and hazard ratio shown.
