# Supplementary material for: Breast cancer stromal clotting activation (Tissue Factor and thrombin): A pre‐invasive phenomena that is prognostic in invasion
Source: Cancer Med. 2020 Jan 21;9(5):1768–78. doi: 10.1002/cam4.2748 (PMC7050075; doi:10.1002/cam4.2748)
Supplement: Supplementary file 5 [file CAM4-9-1768-s005.docx]

**Appendix E:** **Expression of extrinsic clotting pathway markers in normal breast tissue, DCIS and invasive breast cancer

1. Epithelial expression of extrinsic clotting pathway markers in normal, DCIS and invasive breast cancer**

|  | |  | **Normal  n (%)** | **DCIS n (%)** | **Invasive n (%)** | **Pairwise analysis** | ***p*** |
| --- | --- | --- | --- | --- | --- | --- | --- |
| **Tissue Factor** | | |  |  |  |  | 0.24^a^ |
|  | Low expression | | 68 (73) | 25 (74) | 148 (81) |  |  |
|  | High expression | | 25 (27) | 9 (26) | 34 (19) | Normal v DCIS Normal v Inv  Inv v DCIS | 0.96^b^ 0.11^b^ 0.29^b^ |
| **Thrombin** | | |  |  |  |  | **<0.001^a^*** |
|  | Low expression | | 102 (84) | 25 (78) | 103 (55) |  |  |
|  | High expression | | 19 (16) | 7 (22) | 34 (45) | Normal v DCIS Normal v Inv  Inv v DCIS | 0.41^b^ **0.0001^b^* 0.013^b^*** |
| **PAR1** | | |  |  |  |  | 0.32^a^ |
|  | Low expression | | 72 (73) | 20 (63) | 111 (65) |  |  |
|  | High expression | | 27 (27) | 12 (38) | 61 (35) | Normal v DCIS Normal v Inv  Inv v DCIS | 0.27^b^ 0.16^b^* 0.82^b^* |
| **PAR2** | | |  |  |  |  | 0.69^a^ |
|  | Low expression | | 47 (51) | 13 (56) | 97 (52) |  |  |
|  | High expression | | 45 (49) | 12 (44) | 75 (48) | Normal v DCIS Normal v Inv  Inv v DCIS | 0.94^b^ 0.41^b^ 0.68^b^ |
|  | a: Chi-squared test, b: post-hoc Chi-squared test, *:Significant at p<0.05  **Normal** Normal breast tissue **DCIS** Ductal carcinoma in situ **Invasive/Inv** Invasive breast cancer | | | | | | |

**2. Fibroblast expression of extrinsic clotting pathway markers in normal, DCIS and invasive breast cancer**

|  |  | **Normal** | | **DCIS** | **Invasive** | **Pairwise analysis** | ***p*** |
| --- | --- | --- | --- | --- | --- | --- | --- |
| **Tissue Factor** Mean percentage fibroblast expression (SEM) *(n)* | | | 47.1 (3.3) *(190)* | 60.7 (3.7) *(56)* | 72.4 (1.7) *(92)* | Normal vs DCIS Normal vs Inv  Inv vs DCIS | **<0.001^a*^ 0.003^b^* <0.001^b^* 0.004^b^*** |
| **Thrombin** Mean percentage fibroblast expression (SEM) *(n)* | | | 42.1 (2.6) *(134)* | 64.9 (3.9) *(51)* | 62.9 (1.7) *(177)* | Normal vs DCIS Normal vs Inv  Inv vs DCIS | **<0.001^a*^**  **<0.001^b^* <0.001^b^*** 0.62^b^ |
| **PAR1** Mean percentage fibroblast expression (SEM) *(n)* | | | 36.3 (2.7) *(122)* | 51.8 (3.9) *(50)* | 56.9 (1.8) *(173)* | Normal vs DCIS Normal vs Inv  Inv vs DCIS | **<0.001^a*^**  **<0.001^b^* <0.001^b^*** 0.26^b^ |
| **PAR2** Mean percentage fibroblast expression (SEM) *(n)* | | | 44.6 (2.6) *(127)* | 43.4 (3.9) *(50)* | 60.9 (1.8) *(177)* | Normal vs DCIS Normal vs Inv  Inv vs DCIS | **<0.001^a^***  0.75^b^ **<0.001^b^* <0.001^b*^** |
| a: ANOVA, b: post-hoc Least Significant Difference test, *:Significant at p<0.05, SEM; **Normal** Normal breast tissue **DCIS** Ductal carcinoma in situ **Invasive/Inv** Invasive breast cancer | | | | | | | |

**3. Fibroblast expression of extrinsic clotting pathway markers and grade in DCIS**

|  |  |  | **Low grade^1^** | **High grade^2^** | ***p*** |
| --- | --- | --- | --- | --- | --- |
| **Tissue Factor** Mean percentage fibroblast expression (SEM) *(n)* | | | 67.8 (6.2) *(9)* | 55.4 (4.7) *(39)* | 0.12^a^ |
| **Thrombin** Mean percentage fibroblast expression (SEM) *(n)* | | | 61.3 (4.4) *(8)* | 65 (4.4)  *(36)* | 0.7^a^ |
| **PAR1** Mean percentage fibroblast expression (SEM) *(n)* | | | 39 (11.6) *(10)* | 50.3 (5.3) *(36)* | 0.3^a^ |
| **PAR2** Mean percentage fibroblast expression (SEM) *(n)* | | | 55.6 (5.3) *(9)* | 45.0 (4.3) *(36)* | 0.25^a^ |
| 1: Includes low and low/intermediate grade DCIS, 2: Includes intermediate, intermediate/high and high grade DCIS, a: Student’s t-test ***** Significant at p<0.05, | | | | | |

**4. Fibroblast expression of extrinsic clotting pathway markers and ER status in DCIS tumours**

|  |  |  | **ER negative** | **ER positive** | ***p*** | |
| --- | --- | --- | --- | --- | --- | --- |
| **Tissue Factor** Mean percentage  fibroblast expression (SEM) *(n)* | | | 61.5 (6.1) *(13)* | 53.9 (6.5) *(23)* | 0.4^a^ | |
| **Thrombin** Mean percentage  fibroblast expression (SEM) *(n)* | | | 71.8 (5.3) *(17)* | 57 (4.8)  *(27)* | **0.05^a^*** | |
| **PAR1** Mean percentage  fibroblast expression (SEM) *(n)* | | | 55.3 (8.2) *(17)* | 42.2 (6) *(27)* | 0.3^a^ | |
| **PAR2** Mean percentage  fibroblast expression (SEM) *(n)* | | | 41.1 (5.6) *(18)* | 52 (4.5) *(25)* | 0.13^a^ | |
| a: Student’s t-test ***** Significant at p<0.05, | | | | | |  |

**5. Fibroblast expression of extrinsic clotting pathway markers and DCIS tumour size in DCIS tumours**

|  | **DCIS tumour size Coefficient (n)** | ***p*** |
| --- | --- | --- |
| **Tissue Factor** Percentage fibroblast expression | 0.1 *(43)* | 0.7^a^ |
| **Thrombin** Percentage fibroblast expression | 0.5 *(45)* | **0.001^a*^** |
| **PAR1** Percentage fibroblast expression | 0.2 *(45)* | 0.1^a^ |
| **PAR2** Percentage fibroblast expression | -0.1 *(44)* | 0.4^a^ |
| a: Spearman’s correlation coefficient. ***** Significant at p<0.05 | | |

**6. Fibroblast expression of extrinsic clotting pathway markers and invasive grade in invasive cancer**

|  |  | **Grade 1** | **Grade 2** | **Grade 3** | **Pairwise analysis** | | ***p*** |
| --- | --- | --- | --- | --- | --- | --- | --- |
| **Tissue Factor** Mean percentage  fibroblast expression (SEM) *(n)* | | 66.8 (3.6) *(44)* | 68.5 (2.8) *(70)* | 80.6 (2.5) *(66)* | 1 vs 2 1 vs 3 2 vs 3 | | **0.005^a^***  0.56^b^ **0.034^b^* 0.002^b^*** |
| **Thrombin** Mean percentage fibroblast expression (SEM) *(n)* | | 56.1 (4.1) *(45)* | 58.6 (3.1) *(66)* | 71.162.7 (3.4) *(67)* | 1 vs 2 1 vs 3 2 vs 3 | | **0.006^a^***   0.52^b^ **0.003^b^* 0.008^b^*** |
| **PAR1** Mean percentage fibroblast expression (SEM) *(n)* | | 51.4 (4.34) *(42)* | 51.2 (2.9) *(68)* | 66.5 (3.3) *(60)* | 1 vs 2 1 vs 3 2 vs 3 | | **0.001^a*^**   0.96^b^ **0.004^b^* 0.001^b^** |
| **PAR2** Mean percentage fibroblast expression (SEM) *(n)* | | 58.8 (2.4) *42* | 56.4 (2.7) *71* | 69.0 (2.1) *61* | 1 vs 2 1 vs 3 2 vs 3 | | **0.001^a^***   0.52^b^ **0.008^b^* <0.001^b^*** |
| a: ANOVA, b: post-hoc Least Significant Difference test, ***** Significant at p<0.05 | | | | | |  |  |

**7. Fibroblast expression of extrinsic clotting pathway markers and tumour Ki67 expression in invasive cancer**

|  |  |  | **Ki67 <20%** | **Ki67 >20%** | ***p*** |
| --- | --- | --- | --- | --- | --- |
| **Tissue Factor** Mean percentage  fibroblast expression (SEM) *(n)* | | | 67.5 (2.5) *(89)* | 79.3 (2.2) *(86)* | **0.001^a^*** |
| **Thrombin** Mean percentage  fibroblast expression (SEM) *(n)* | | | 56.6 (2.9) *(88)* | 69.5 (2.8)  *(85)* | **0.002^a^*** |
| **PAR1** Mean percentage  fibroblast expression (SEM) *(n)* | | | 49.1 (2.8) *(85)* | 64.0 (2.7) *(82)* | **<0.001^a^*** |
| **PAR2** Mean percentage  fibroblast expression (SEM) *(n)* | | | 57.1 (2.2) *(86)* | 66.2 (1.9) *(83)* | **0.002^a^*** |
| a: ANOVA, b: post-hoc Least Significant Difference test, ***** Significant at p<0.05, | | | | | |

**8. Fibroblast expression of extrinsic clotting pathway markers and ER status in invasive cancer**

|  |  |  | **ER negative** | **ER positive** | ***p*** |
| --- | --- | --- | --- | --- | --- |
| **Tissue Factor** Mean percentage  fibroblast expression (SEM) *(n)* | | | 80.3 (3.5) *(39)* | 71.6 (1.9) *(142)* | 0.04^a^** |
| **Thrombin** Mean percentage  fibroblast expression (SEM) *(n)* | | | 65 (4.2) *(36)* | 61.3 (2.3) *(137)* | 0.15^a^ |
| **PAR1** Mean percentage  fibroblast expression (SEM) *(n)* | | | 69.7 (4.3) *(36)* | 53.3 (2.2) *(135)* | **0.001^a^*** |
| **PAR2** Mean percentage  fibroblast expression (SEM) *(n)* | | | 66.2 (3.5) *(34)* | 60.4 (1.6) *(141)* | 0.13^a^ |
| a: ANOVA, b: post-hoc Least Significant Difference test, ***** Significant at p<0.05 | | | | | |

**9. Fibroblast expression of extrinsic clotting pathway markers and HER2 status in invasive cancer**

|  |  |  | **HER2 negative** | **HER2 positive** | ***p*** |
| --- | --- | --- | --- | --- | --- |
| **Tissue Factor** Mean percentage  fibroblast expression (SEM) *(n)* | | | 72.1 (1.8) *(152)* | 84.8 (4.1) *(23)* | **0.01^a^*** |
| **Thrombin** Mean percentage  fibroblast expression (SEM) *(n)* | | | 61.1 (2.3)  *(144)* | 77.0 (4.2) *(23)* | **0.002^a^*** |
| **PAR1** Mean percentage  fibroblast expression (SEM) *(n)* | | | 56.6 (2.2) *(152)* | 57.4 (5.6) *(19)* | 0.99^a^ |
| **PAR2** Mean percentage  fibroblast expression (SEM) *(n)* | | | 60.0 (1.6) *(157)* | 74.4 (3.0) *(18)* | **0.001^a^*** |
| a: ANOVA, b: post-hoc Least Significant Difference test, ***** Significant at p<0.05 | | | | | |

**10. Fibroblast expression of extrinsic clotting pathway markers and invasive tumour size**

|  |  |  | **Small tumour^1^** | **Large tumour^2^** | ***p*** |
| --- | --- | --- | --- | --- | --- |
| **Tissue Factor** Mean percentage  fibroblast expression (SEM) *(n)* | | | 73.3 (2.42)  *(93)* | 73.5 (2.4) *(88)* | 0.34^a^* |
| **Thrombin** Mean percentage  fibroblast expression (SEM) *(n)* | | | 61.0 (2.9) *(89)* | 64.1 (2.9)  *(84)* | 0.34^a^* |
| **PAR1** Mean percentage  fibroblast expression (SEM) *(n)* | | | 55.2 (2.9) *(88)* | 58.1 (2.9) *(82)* | 0.40^a^ |
| **PAR2** Mean percentage  fibroblast expression (SEM) *(n)* | | | 59.3 (2.1) *(91)* | 63.7 (1.9) *(83)* | 0.14^a^ |
| ^1^: <median (13mm in CHAMPion study), ^2^: >median (13mm in CHAMPion study and), ^a^: ANOVA, ^b^: post-hoc Least Significant Difference test, ***** Significant at p<0.05 | | | | | |

**11. Stromal fibroblast expression of extrinsic clotting pathway markers and lymph node status in invasive cancer**

|  |  |  | **Lymph node negative** | **Lymph node positive** | ***p*** |
| --- | --- | --- | --- | --- | --- |
| **Tissue Factor** Mean percentage  fibroblast expression (SEM) *(n)* | | | 71.9 (2.1)  *(122)* | 76.4 (2.7) *(53)* | 0.23^a^ |
| **Thrombin** Mean percentage  fibroblast expression (SEM) *(n)* | | | 60.5 (2.6) *(134)* | 64.1 (2.9)  *(49)* | 0.38^a^ |
| **PAR1** Mean percentage  fibroblast expression (SEM) *(n)* | | | 53.7 (2.4) *(114)* | 66.2 (3.8) *(50)* | 0.005^a^* |
| **PAR2** Mean percentage  fibroblast expression (SEM) *(n)* | | | 60.2 (1.9)  *(133)* | 66.1 (2.2) *(49)* | 0.08^a^ |
| a: ANOVA, b: post-hoc Least Significant Difference test, ***** Significant at p<0.05 | | | | | |

**12. Fibroblast expression of extrinsic pathway markers and intrinsic molecular subtype in invasive cancer**

|  |  | **Luminal A** | **Luminal B** | **HER2-OE** | **Basal** | **Pair-wise analysis** | ***p*** |
| --- | --- | --- | --- | --- | --- | --- | --- |
| **Tissue Factor** Mean percentage fibroblast expression (SEM) *(n)* | | 69.0 (2.7) *(73)* | 74.3 (2.8) *(68)* | 88.2 (3.8) *(11)* | 77.1 (4.6) *(28)* | L-A vs L-B L-A vs HER L-A vs Bas | **0.04^a^***  0.65^b^ 0.01^b^* 0.10^b^ |
| **Thrombin** Mean percentage fibroblast expression (SEM) *(n)* | | 55.8 (3.2) *(73)* | 66.6 (3.3) *(65)* | 75.5 (7.9) *(11)* | 65.6 (7.9) *(25)* | L-A vs L-B L-A vs HER L-A vs Bas | **0.03^a^***   0.02^b^ 0.02^b^ 0.12^b^ |
| **PAR1** Mean percentage fibroblast expression (SEM) *(n)* | | 52.7 (3.1) *(73)* | 53.9 (3.1) *(62)* | 58.9 (9.6) *(9)* | 73.3 (4.7) *(27)* | L-A vs L-B L-A vs HER L-A vs Bas | **0.004^a*^**   0.79^b^ 0.49 **<0.001^b^*** |
| **PAR2** Mean percentage fibroblast expression (SEM) *(n)* | | 58.9 (2.3) *(73)* | 56.4 (2.7) *(68)* | 69.0 (2.1) *(8)* | 69.0 (2.1) *(26)* | L-A vs L-B L-A vs Bas L-A vs HER | 0.11^a^   0.36^b^ **0.02^b^*** 0.35^b^ |
| a: ANOVA, b: post-hoc Least Significant Difference test, ***** Significant at p<0.05, q-values not calculated, **L-A** Luminal A **L-B** Luminal B **HER** HER2-Overexpressing **Bas** Basal | | | | | | | |
