# Supplementary material for: Breast cancer stromal clotting activation (Tissue Factor and thrombin): A pre‐invasive phenomena that is prognostic in invasion
Source: Cancer Med. 2020 Jan 21;9(5):1768–78. doi: 10.1002/cam4.2748 (PMC7050075; doi:10.1002/cam4.2748)
Supplement: Supplementary file 6 [file CAM4-9-1768-s006.docx]

**Appendix F:** **Supplementary pre-operative plasma data**

**Table F.1. Pre-operative plasma extrinsic clotting pathway markers in DCIS and invasive breast cancer patients**

| **Plasma marker** | **DCIS** | **Invasive** | ***p*** |
| --- | --- | --- | --- |
| **TF** pg/ml, geometric mean (95% CI) *(n)* | 184(164-206) *(56)* | 177 (164-191) *(233)* | 0.68^a^ |
| **TAT** ng/ml, geometric mean (95% CI) *(n)* | 3.3 (2.6-4.1) *(56)* | 3.8 (3.3-4.4) *(232)* | 0.58^a^ |
| **D-dimer** ng/ml, geometric mean (95% CI) *(n)* | 402 (351-462) *(56)* | 443 (405-485) *(239)* | 0.3^b^ |
| a: Student’s t-test b: Analysis of covariance (ANCOVA, age, body mass index and hypertension as co-variates). ***:** Significant at p<0.05, **TF** Tissue Factor **TAT** Thrombin-antithrombin **DCIS** Ductal carcinoma in-situ **Invasive/Inv** Invasive breast cancer | | | |

**Table F.2. Pre-operative plasma extrinsic clotting pathway markers and lymph node status in invasive breast cancer patients**

| **Plasma marker** | **Lymph node negative (All invasive cancers)** | **Lymph node positive (All invasive cancers)** | ***p*** |
| --- | --- | --- | --- |
| **TF** pg/ml, geometric mean (95% CI) *(n)* | 170 (156-185) *(157)* | 193(163-228) *(66)* | 0.45^a^ |
| **TAT** ng/ml, geometric mean (95% CI) *(n)* | 3.7 (3.1-4.4) *(156)* | 4.7 (3.4-6.5) *(66)* | 0.23^a^ |
| **D-dimer** ng/ml, geometric mean  (95% CI) *(n)* | 432 (391-476) *(171)* | 507 (411-625) *(68)* | 0.004^b^ |
| a: Student’s t-test b: Analysis of covariance (ANCOVA, age, body mass index and hypertension as co-variates). ***:** Significant at p<0.05, **TF** Tissue Factor **TAT** Thrombin-antithrombin | | | |

**Table F.3. Pre-operative plasma extrinsic clotting pathway markers and ER status in invasive breast cancer patients**

|  |  |  | **ER negative** | **ER positive** | ***p*** |
| --- | --- | --- | --- | --- | --- |
| **TF** pg/ml, geometric mean (95% CI) *(n)* | | | 197 (167-232) *(43)* | 173 (159-188) *(189)* | 0.19^a^ |
| **TAT** ng/ml, geometric mean (95% CI) *(n)* | | | 4.0 (2.6-6.2) *(43)* | 3.8 (3.2-4.4) *(188)* | 0.77^a^ |
| **D-dimer** ng/ml, geometric mean  (95% CI) *(n)* | | | 496 (398-617) *(44)* | 436 (395-482) *(194)* | 0.28^b^ |
| a: Student’s t-test b: Analysis of covariance (ANCOVA, age, body mass index [BMI] and hypertension as co-variates), ***:** Significant at p<0.05, **TF** Tissue Factor **TAT** Thrombin-antithrombin | | | | | |

**Table F.4. Pre-operative plasma extrinsic clotting pathway markers and HER2 status in invasive breast cancer patients**

|  |  |  | **HER2 negative** | **HER2 positive** | ***p*** |
| --- | --- | --- | --- | --- | --- |
| **TF** pg/ml, geometric mean (95% CI) *(n)* | | | 174 (160-189) *(197)* | 189 (154-232) *(26)* | 0.48^a^ |
| **TAT** ng/ml, geometric mean (95% CI) *(n)* | | | 3.9 (3.3-4.7) *(196)* | 3.7 (2.6-5.1) *(26)* | 0.76^a^ |
| **D-dimer** ng/ml, geometric mean  (95% CI) *(n)* | | | 445 (402-491) *(203)* | 506 (394-649) *(27)* | 0.38^b^ |
| a: Student’s t-test, b: Analysis of covariance (ANCOVA, age, BMI and hypertension as co-variates), ***:** Significant at p<0.05, **TF** Tissue Factor **TAT** Thrombin-antithrombin | | | | | |

**Table F.5. Pre-operative plasma extrinsic clotting pathway markers and Ki67 expression in invasive breast cancer patients**

|  |  |  | **Ki67 <20%** | **Ki67 >20%** | ***p*** |
| --- | --- | --- | --- | --- | --- |
| **TF** pg/ml, geometric mean (95% CI) *(n)* | | | 166 (149-185) *(120)* | 182 (163-203) *(101)* | 0.27^a^ |
| **TAT** ng/ml, geometric mean (95% CI) *(n)* | | | 4.2 (3.4-5.1) *(120)* | 3.7 (2.9-4.7) *(100)* | 0.40^a^ |
| **D-dimer** ng/ml, geometric mean (95% CI) *(n)* | | | 451 (396-513) *(126)* | 445 (385-514) *(101)* | 0.74^b^ |
| a: Student’s t-test, b: Analysis of covariance (ANCOVA, age, BMI and hypertension as co-variates) ***:** Significant at p<0.05, **TF** Tissue Factor **TAT** Thrombin-antithrombin | | | | | |

**Table F.6. Pre-operative plasma extrinsic clotting pathway markers and invasive grade in invasive breast cancer patients**

|  | **Grade 1** | **Grade 2** | **Grade 3** | **Pairwise analysis** | ***p*** |
| --- | --- | --- | --- | --- | --- |
| **TF** pg/ml, geometric mean (95% CI) *(n)* | 186 (165-210) *(55)* | 159 (140-182) *(99)* | 190 (169-214) *(76)* | 1 vs 2 1 vs 3  2 vs 3 | 0.09^a^  0.11^b^ 0.73^b^ 0.03^b^** |
| **TAT** ng/ml, geometric mean (95% CI) *(n)* | 4.6 (3.4-6.2) *(55)* | 3.5 (2.8-4.4) *(99)* | 3.9 (2.9-5.2) *(75)* | 1 vs 2 1 vs 3  2 vs 3 | 0.42^a^  0.226^b^ 0.562^b^ 0.504^b^ |
| **D-dimer** ng/ml, geometric mean  (95% CI) *(n)* | 500 (410-608) *(60)* | 419 (373-470) *(102)* | 442 (367-532) *(76)* | 1 vs 2 1 vs 3  2 vs 3 | 0.15^c^  0.06^d^ 0.15^d^ 0.65^d^ |
| a: ANOVA, b: post-hoc Least Significant Difference test, c: Analysis of covariance (ANCOVA, age, body mass index and hypertension as co-variates) d:post-hoc ANCOVA, ***:** Significant at p<0. | | | | | |

**Table F.7. Pre-operative plasma extrinsic clotting pathway markers and invasive tumour size in invasive breast cancer**

| **Clotting pathway marker (n)** | **Invasive tumour size  Coefficient (n)** | ***p*** |
| --- | --- | --- |
| TF pg/ml  *(231)* | 0.08 *(231)* | 0.23^a^ |
| TAT µg/ml  *(232)* | 0.04 *(232)* | 0.49 ^a^ |
| D-dimer ng/ml  *(231)* | 0.01 *(231)* | 0.99 ^a^  0.16^b^ |
| a: Spearman’s correlation coefficient. b: Analysis of covariance (ANCOVA, age, body mass index and hypertension as co-variates). ***** Significant at p<0.05, **TF** Tissue Factor **TAT** Thrombin-antithrombin | | |

**Table F.8 Pre-operative plasma extrinsic clotting pathway markers and presence of lymphovascular invasion in invasive breast cancer patients**

| **Plasma marker** | **Lymphovascular invasion absent (All invasive cancers)** | **Lymphovascular invasion possible (All invasive cancers)** | **Lymphovascular invasion present (All invasive cancers)** | ***p*** |
| --- | --- | --- | --- | --- |
| **TF** pg/ml, geometric mean (95% CI) *(n)* | 177 (164-190) *(171)* | 141 (76-263) *(10)* | 182(149-224) *(66)* | 0.44^a^ |
| **TAT** ng/ml, geometric mean (95% CI) *(n)* | 3.7 (3.1-4.3) *(169)* | 3.1 (1.5-6.2) *(10)* | 4.1 (2.9-5.8) *(51)* | 0.68^a^ |
| **D-dimer** ng/ml, geometric mean  (95% CI) *(n)* | 462 (419-508) *(175)* | 373 (193-720) *(11)* | 440 (351-551) *(53)* | 0.59^b^ |

a: ANOVA, b: Analysis of covariance (ANCOVA, age, body mass index and hypertension as co-variates) d:post-hoc ANCOVA,


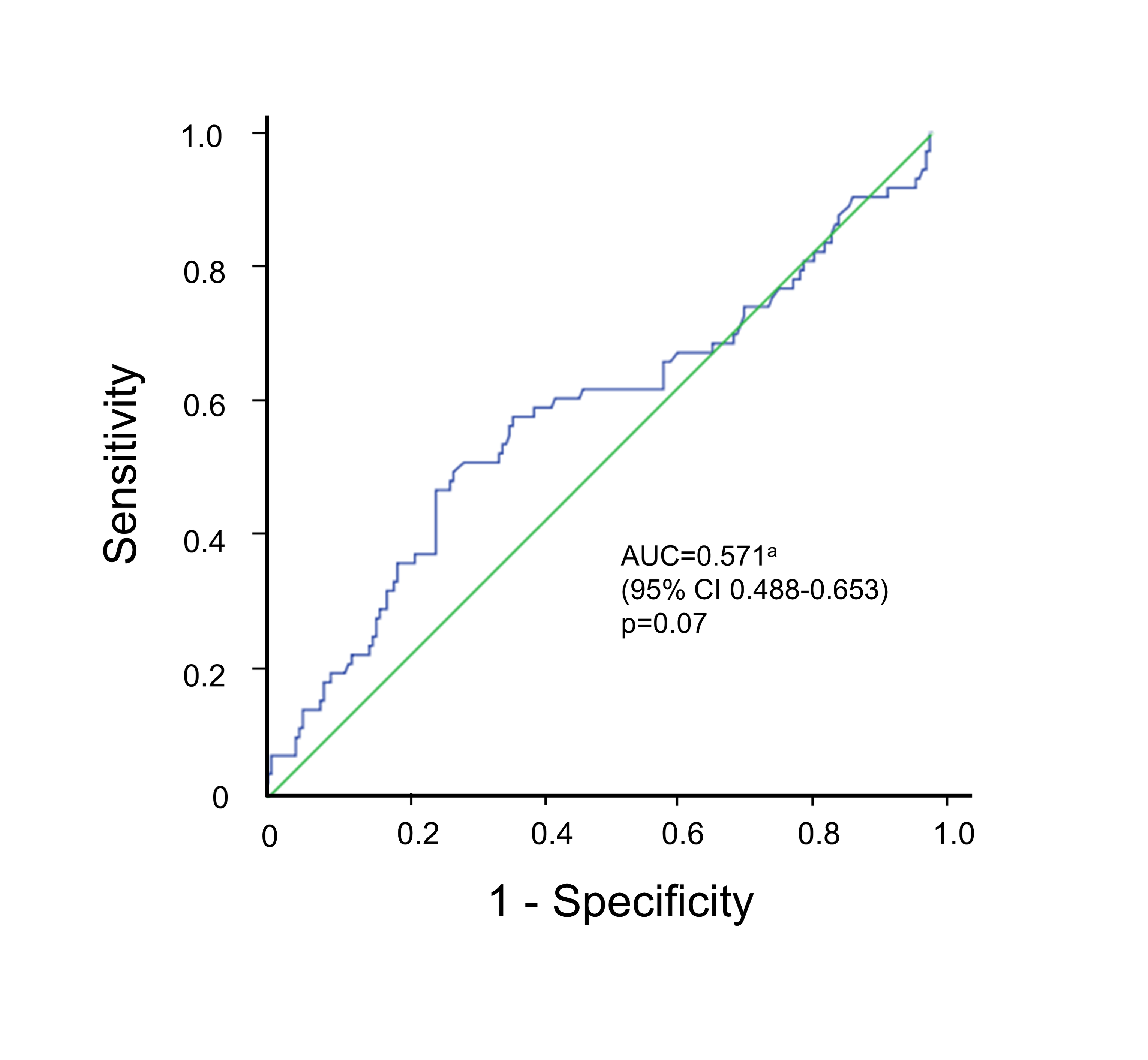

**Figure F.1. Receiver operating characteristics (ROC) curve for raised d-dimer (>559 ng/ml) in discriminating between lymph node negative and lymph node positive invasive breast cancer patients.** a: Null hypothesis AUC (area under the curve) =0.5. n=239 (CHAMPion invasive cancers). D-dimer cut-off of 559 ng/ml had the greatest sensitivity (46%) and specificity (72%) to identify lymph node positive patients. Clinicopathological variables known to be associated with lymph node metastasis were inserted into a univariate binary logistic regression model along with D-dimer (dichotomised into above and below 559 ng/ml) and other variables known to correlate with increased D-dimer (age, BMI and hypertension). Invasive grade, invasive tumour size, Ki67 expression, ER status and a raised D-dimer were significantly associated with lymph node metastasis and were inserted into multivariate (backward conditional) binary logistic regression model. On multivariate analysis, including D-dimer above 559 ng/ml was a significant predictor of node positivity (odds ratio 2.53, 95% CI: 1.33-4.83, p=0.005).
